# Cancer-associated fibroblast-induced lncRNA UPK1A-AS1 confers platinum resistance in pancreatic cancer via efficient double-strand break repair

**DOI:** 10.1038/s41388-022-02253-6

**Published:** 2022-03-09

**Authors:** Xiang Zhang, Shangyou Zheng, Chonghui Hu, Guolin Li, Hongcao Lin, Renpeng Xia, Yuancheng Ye, Rihua He, Zhihua Li, Qing Lin, Rufu Chen, Quanbo Zhou

**Affiliations:** 1 Guangdong Provincial Key Laboratory of Malignant Tumor Epigenetics and Gene Regulation, Sun Yat-Sen Memorial Hospital, Sun Yat-Sen University, Guangzhou, 510120 Guangdong People’s Republic of China; 2 Department of Pancreatobiliary Surgery, Sun Yat-sen Memorial Hospital, Sun Yat-sen University, Guangzhou, 510120 Guangdong People’s Republic of China; 3 Department of Pancreas Center, Department of General Surgery, Guangdong Provincial People’s Hospital, Guangdong Academy of Medical Sciences, Guangzhou, 510080 Guangdong People’s Republic of China; 4 The Second School of Clinical Medicine, Southern Medical University, Guangzhou, 510515 Guangdong People’s Republic of China; 5 Guangdong cardiovascular Institute, Guangzhou, 510080 Guangdong People’s Republic of China; 6 Department of Hepatobiliary, Pancreatic and Splenic surgery, The Sixth Affiliated Hospital of Sun Yat-sen University, Guangzhou, 510655 Guangdong People’s Republic of China; 7 General Surgery of Shenshan Medical Center, Memorial Hospital of Sun Yat-sen University, Shanwei, 516600 Guangdong People’s Republic of China; 8 Department of Neonatal/General Surgery, Hunan Children’s Hospital, Changsha, 410007 Hunan People’s Republic of China; 9 Department of Oncology, Sun Yat-sen Memorial Hospital, Sun Yat-sen University, Guangzhou, 510120 Guangdong People’s Republic of China; 10 School of medicine, South China University of Technology, Guangzhou, 510006 Guangdong People’s Republic of China

**Keywords:** Cancer microenvironment, Cancer therapeutic resistance

## Abstract

The tumor stroma of pancreatic ductal adenocarcinoma (PDAC) is characterized by an abundant and heterogeneous population of cancer-associated fibroblasts (CAFs), which are critically involved in chemoresistance. However, the underlying mechanism of CAFs in chemoresistance is unclear. Here, we show that CAF^R^, a CAF subset derived from platinum-resistant PDAC patients, assumes an iCAF phenotype and produces more IL8 than CAF^S^ isolated from platinum-sensitive PDAC patients. CAF^R^-derived IL8 promotes oxaliplatin chemoresistance in PDAC. Based on long noncoding RNA (lncRNA) profiling in tumor cells incubated with CAF-CM, we found that UPK1A-AS1, whose expression is directly induced by IL8/NF-kappa B signaling, functions as a chemoresistance-promoting lncRNA and is critical for active IL8-induced oxaliplatin resistance. Impressively, blocking the activation of UPK1A-AS1 expression increases the oxaliplatin sensitivity of tumor cells in vivo. Mechanistically, UPK1A-AS1 strengthens the interaction between Ku70 and Ku80 to facilitate nonhomologous end joining (NHEJ), thereby enhancing DNA double-strand break (DSB) repair. Clinically, UPK1A-AS1 expression is positively correlated with IL8 expression, a poor chemotherapeutic response and a shorter progression-free survival (PFS) time in advanced PDAC patients. Collectively, our study reveals a lncRNA-mediated mechanism of CAF-derived paracrine IL8-dependent oxaliplatin resistance and highlights UPK1A-AS1 as a potential therapeutic target.

## Introduction

Pancreatic ductal adenocarcinoma (PDAC) is an extremely lethal disease despite the application of multiple therapeutic strategies [1]. Targeting the tumor microenvironment (TME) is a very popular strategy in cancer therapy, while strategies aiming to degrade the desmoplastic stroma have been generally disappointing due to the highly complex interplay between cancer cells and the TME [2, 3]. During chemotherapy or radiotherapy, cancer cells adapt to selective pressure by self-mutation or transformation [4], but the ability to modulate various components of the TME in response to chemo- and radiotherapy is incompletely elucidated.

Cancer-associated fibroblasts (CAFs), a major component of the stroma that undergoes transformation and exhibits heterogeneity during cancer evolution, can perform both tumor-promoting and tumor-suppressive or homeostatic functions in PDAC [5]. Different CAF populations with distinct phenotypes, epigenetic characteristics, immunogenicity, and transduction signatures have been recently identified in pancreatic cancers [6–8], and reshaping the CAF population can improve the efficacy of current standard therapies [9, 10]. Previously, researchers identified two types of CAFs using mouse and human pancreatic cancer models. One subgroup named inflammatory CAFs (iCAFs) is mainly characterized by the secretion of inflammatory factors (such as IL-6, LIF, IL-1, etc.) and is relatively far from tumor cells, while the other subpopulation, named myofibroblastic CAFs (myCAFs), expresses characteristic proteins, such as a-SMA, TGFβ, and ECM, and is distributed adjacent to tumor cells [9, 11]. Furthermore, recently, through a single-cell analysis, a subgroup of CAFs (named antigen-presenting CAFs (apCAFs)) that mainly function to mediate the immune response of pancreatic cancer has also been found and is mainly characterized by the expression of MHC class II molecules and CD74 [7]. Therefore, to precisely target the CAF subpopulations that support tumor growth, it is necessary to enhance our understanding of their functions and mechanisms.

Given that over 80% of PDACs are nonresectable at the time of diagnosis [12], chemotherapy is the major treatment option for most PDAC patients. However, drug resistance is an inevitable problem that arises in almost all patients [13]. Recently, chemotherapeutic resistance mediated by the TME has attracted increasing attention. Although increasing evidence has demonstrated the associations between the CAF subtypes and drug resistance [13, 14], neither the CAF subpopulation responsible for drug sensitivity nor its mechanism has been identified. Existing evidence shows that CAFs confer chemotherapeutic resistance by inducing a desmoplastic reaction that interferes with drug delivery, and the complex mechanisms of cytokine, chemokine, and growth factor secretion are also involved in the drug response [15]. Recently, platinum-based chemotherapy has gradually attracted attention because patients with PDAC and germline or somatic pathogenic variants in BRCA or PALB2 are highly sensitive to platinum chemotherapies and poly (adenosine diphosphate-ribose) polymerase (PARP) inhibitor (PARPi) therapy [16, 17]. Platinum molecules crosslink with the purine bases of DNA and interfere with DNA replication and transcription, thereby triggering DNA damage and degradation [18]. In patients with pancreatic cancer, homologous recombination (HR) deficiency may lead to the inability to effectively repair double-strand breaks (DSBs), and these patients are particularly sensitive to platinum-based chemotherapy [19]. However, only 21% of pancreatic cancer patients are sensitive to platinum-based chemotherapy, and the objective response rate to platinum-based regimens is only ~50%, even among pancreatic cancer patients with germline BRCA or PALB2 mutations [20]. Regarding the mechanisms of platinum resistance, possible causes, such as secondary mutations restoring DNA repair pathways, factors restricting the crosslinking of a drug molecule with its target, and pathways eliminating damage from the target, have been studied [21]. The CAF-mediated modulation of platinum resistance via the prevention of sufficient drug delivery plays a key role in modulating platinum resistance in PDAC [22]. Recently, Natalia et al. demonstrated that the blockade of TGFβ-activated kinase 1 (TAK1) and TGFBR1 removes the chemoprotection conferred by proinflammatory factor-mediated CAF activation, thereby rendering tumor cells more sensitive to platinum therapy in colorectal cancer [23]. In addition, Wang et al. showed that CAFs mediated a nongenetic mechanism by regulating thiol metabolism to confer cisplatin resistance in ovarian cancer [24]. However, the precise role of CAFs in the mediation of platinum resistance in pancreatic cancer tissues remains to be elucidated.

Long noncoding RNAs (lncRNAs) constitute a heterogeneous class of transcripts longer than 200 nucleotides and are involved in multiple cellular processes by interacting with DNA, chromatin, proteins and RNAs [25]. Recently, substantial evidence has indicated that lncRNAs are involved in the chemoresistance of pancreatic cancer cells [26]; for instance, a report showed that the lncRNA HOTTIP confers cisplatin resistance on PDAC cells by regulating miR-137 expression [27]. Furthermore, Deng et al. revealed that the lncRNA CCAL derived from CAFs drives oxaliplatin resistance in colorectal cancer cells by modulating the mRNA stability of β-catenin. Nevertheless, the roles of CAF-derived lncRNAs in inducing platinum resistance in pancreatic cancer are poorly understood.

Here, we sought to gain a better understanding of the mechanisms by which CAFs support platinum resistance in PDAC cells. Furthermore, we studied the role of CAF-induced lncRNAs associated with platinum resistance and explored the underlying mechanisms while aiming to develop rational strategies that combine conventional cytotoxic agents and selectively target the protumorigenic functions of CAFs and lncRNAs.

## Results

### CAFs were correlated with platinum resistance in PDAC

**Fig. 1:**
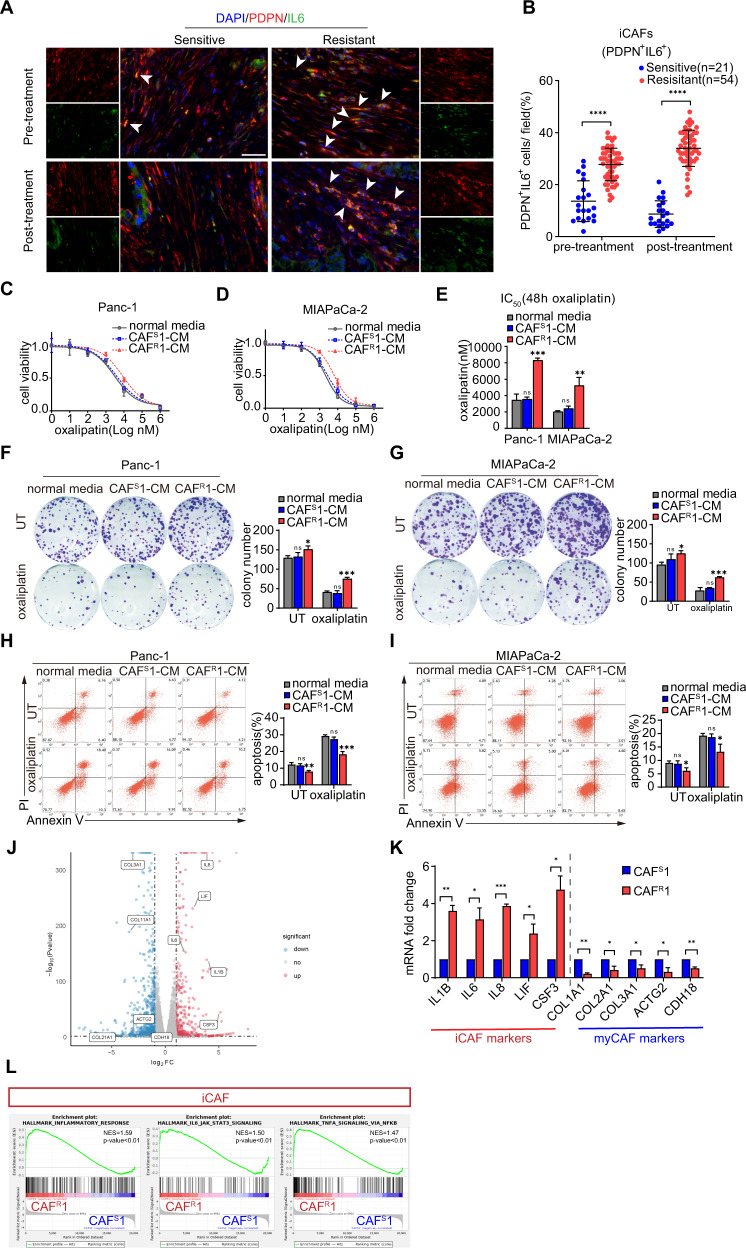
CAFs were correlated with a poor survival and platinum resistance in pancreatic cancer patients. **A** Representative immunofluorescence images of advanced pancreatic cancer biopsies before and after chemotherapy. Arrows indicate iCAFs: PDPN ^+^IL6^+^. Scale bar = 50 μm. Quantification of the presence of iCAFs (**B**). Panc-1 and MIAPaCa-2 cells were cultured under CAF^S^1-CM or CAF^R^1-CM for 3 days and then subjected to the indicated experiments. **C–E** Cells were treated with oxaliplatin for 48 h. Cell viability was measured by CCK-8 and the IC50 value was calculated. **F**, **G** Colony formation assay and (**H**, **I**) flow cytometry apoptosis analyses were performed to evaluate the chemoresistance of pancreatic cancer cells in each group. **J** Volcano plot of differentially expressed genes from the mRNA transcriptomes of CAF^S^1-CM and CAF^R^1-CM. Three biological replicates of each CAF cell line were used for the RNA sequencing. **K** Expression level of specific genes in CAF^S^1-CM and CAF^R^1-CM. **L** GSEA of significantly upregulated pathways in CAF^R^1 compared with CAF^S^1. The results are presented as the mean ± SD of three technical replicates. *P < 0.05; **P < 0.01; ***P < 0.001, ns no significance.

To investigate the inherent heterogeneity of CAFs in chemoresistance, we adopted a clinical model of platinum-based chemotherapy for advanced pancreatic cancer to monitor the diverse response of tumors to drugs. Paired tumor samples were obtained from 75 PDAC patients before and after chemotherapy. The baseline characteristics of the enrolled patients were showed in Table 1. Then, an immunofluorescence analysis of different CAF populations was performed (iCAF: PDPNα ^+^IL6^+^; myCAF: PDPN^+^αSMA^+^; apCAF: PDPN^+^MHCII^+^) [9, 22] (Figs. 1A, S1A and C). The results showed that the proportion of iCAFs was significantly increased in the tissue from the chemoresistant patients before and after chemotherapy (Fig. 1B), while the amounts of myCAFs and apCAFs did not differ between the chemoresistant and chemosensitive patients (Fig. S1B and D).

**Table 1:** Baseline characteristics of the patients.

Characteristic	Chemosensitive patients (*n* = 21)	Chemoresistant patients (*n* = 54)
Age at randomization — yr		
Median	53	58
Range	41–70	37–82
Male sex — no (%)	10 (48)	36 (67)
TNM stage — no (%)		
III	7 (33)	23 (43)
IV	14 (67)	31 (57)
First-line platinum-based chemotherapy — no. (%)		
mFOLFIRINOX	18 (86)	42 (78)
GemOx	1 (5)	7 (13)
GP	2 (10)	5 (9)
Rounds of mFOLFIRINOX		
Median	11	3.5
Range	6–12	1–6
Rounds of GemOx		
Median	8	3
Range	8	2–4
Rounds of GP		
Median	7	4
Range	6–8	3–4
Germline BRCA or PALB2 mutation — no. (%)		
BRCA1	0 (0)	0 (0)
BRCA2	2 (10)	0 (0)
PALB2	2 (10)	0 (0)

To determine whether CAFs contribute to chemoresistance, CAFs were isolated from PDAC tissues collected before platinum-based chemotherapy. All CAF cell lines were identified by FSP-1 immunofluorescence, and tested negative for EpCAM, CD31 and CD45 by a flow cytometry analysis (Fig. S1E, F). Then pancreatic cancer cell lines (Panc-1 and MIAPaCa-2) were treated with conditioned medium from CAFs isolated from two different chemoresistant patients (CAF^R^1-CM and CAF^R^2-CM) and CAFs isolated from two different chemosensitive patients (CAF^S^1-CM and CAF^S^2-CM). Interestingly, compared to the CAF^S^-CM treatment, the CAF^R^-CM treatment contributed to an approximately twofold increase in the IC50 value of oxaliplatin in the tumor cells (Figs. 1C–E and S2A–C). Consistent with this result, upon the treatment with CAF^R^-CM, colony formation was promoted in the tumor cells, and more colonies survived after the oxaliplatin exposure (Figs. 1F, G and S2D, E). To further confirm the contribution of CAFs to oxaliplatin-induced cell death, a flow cytometry-based Annexin-V/propidium iodide (PI) apoptosis assay was performed. Apoptosis of tumor cells was suppressed under the culture with CAF^R^-CM, and the apoptosis-inducing effect of oxaliplatin on the tumor cells was abrogated by the CAF^R^-CM treatment compared with normal medium or the CAF^S^-CM treatment (Figs. 1H, I and S2F, G). Taken together, our data showed that CAF^R^-CM conferred oxaliplatin resistance on pancreatic tumor cells.

Subsequently, to determine whether CAF^R^ assumes an iCAF phenotype, we performed next-generation RNA sequence (GSE192907) to compare the transcriptome differences between CAF^R^1 and CAF^S^1. We found a cluster of genes differentially expressed in either CAF^R^1 or CAF^S^1. Specifically, cytokines, such as IL1, IL6, IL8, LIF and CSF3 were uniquely upregulated in CAF^R^1. However, the smooth muscle gene ACTG2 and various collagen genes were upregulated in CAF^S^1 (Fig. 1J, K). Furthermore, a gene set enrichment analysis (GSEA) of CAF^R^1 compared with CAF^S^1 confirmed the upregulation of the inflammatory response pathway and IL6-JAK-STAT3 signaling pathway, which is consistent with previous study (Fig. 1L) [9]. In addition, we performed PCR and sequencing to detect whether the most frequent mutations in PDAC exist in these CAF cell lines. The results showed that there were no mutations of KRAS, TP53, and SMAD4 and homozygous deletion of CDKN2A in the CAF cell lines (Fig. S2H, I).

### Paracrine IL8 was essential for CAF^R^-induced oxaliplatin resistance in tumor cells

**Fig. 2:**
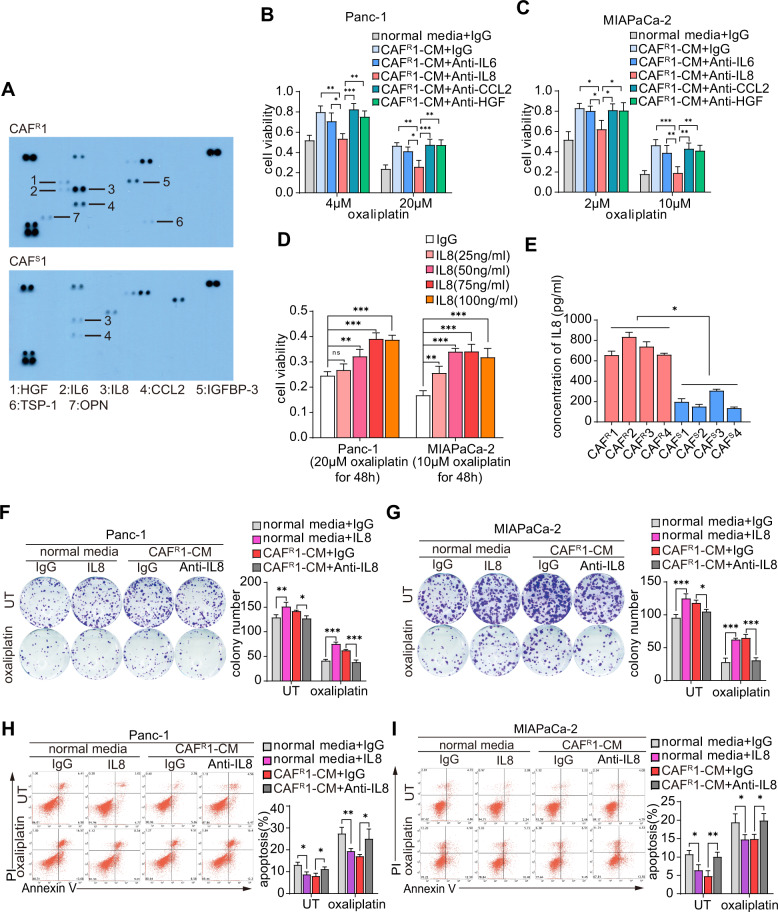
Paracrine IL8 was essential for CAF^R^-induced oxaliplatin resistance in tumor cells. **A** Cytokine antibody array of CAF^S^1-CM or CAF^R^1-CM. CAF^R^1-CM with different neutralizing antibodies was used to culture Panc-1 (**B**) and MIAPaCa-2 (**C**) cells for 3 days. After 48 h of oxaliplatin exposure, cell viability was measured by CCK8. **D** Panc-1 and MIAPaCa-2 cells were cultured with different concentrations of human recombinant IL8 for 3 days and subsequently treated with oxaliplatin for 48 h. Cell viability was measured by CCK8. **E** ELISA analysis of the IL-8 levels in CAF^S^-CM and CAF^R^-CM of four different chemosensitive and chemoresistant CAF cell lines. Panc-1 and MIAPaCa-2 cells were cultured with IL8 (100 ng/ml) or CAF^R^1-CM with an anti-IL8 neutralizing antibody (250 ng/ml) for 3 days. Colony formation assay (**F**, **G**) and flow cytometry apoptosis analysis (**H**, **I**) were performed to evaluate the chemoresistance of pancreatic cancer cells in each group. The results are presented as the mean ± SD of three technical replicates. *P < 0.05; **P < 0.01; ***P < 0.001; ns no significance.

To reveal the mechanism by which CAFs contribute to chemoresistance, we compared the cytokine profiles of CAF^R^1-CM and CAF^S^1-CM by using a human cytokine antibody array and identified a panel of cytokines with high levels in CAF^R^1-CM (Fig. 2A). Among these cytokines, HGF, IL6, IL8 and CCL2 have been documented to induce chemoresistance [28–31]. By adding neutralizing antibodies against each candidate cytokine, we further identified the potential cytokines responsible for inducing oxaliplatin resistance. The cell viability assays revealed that the anti-IL8 antibody but not the other three neutralizing antibodies against HGF, IL6 and CCL2 successfully abrogated the ability of CAF^R^1-CM and CAF^R^2-CM to induce oxaliplatin resistance (Figs. 2B, C and S3A, B). Moreover, human recombinant IL8 protected tumor cells against oxaliplatin in a dose-dependent manner (Fig. 2D). An ELISA was further performed to measure the concentration of IL8 in CAF^R^-CM and CAF^S^-CM extracted from 4 different chemoresistant and chemosensitive patients. As expected, CAF^R^s secreted much more IL8 than CAF^S^s (Fig. 2E). Furthermore, in the colony formation assays, the disruption of IL8 signaling by the addition of anti-IL8 antibodies reduced tumor cell colony formation and survival, which were promoted by CAF^R^-CM (Figs. 2F, G and S3C, D). In addition, anti-IL8 antibodies increased oxaliplatin-induced tumor cell apoptosis, which was repressed by CAF^R^-CM (Figs. 2H–I and S3E, F).

Collectively, these data suggest that IL8 was predominantly produced by CAF^R^s and that the functional disruption of IL8 abrogated CAF^R^-induced oxaliplatin resistance in tumor cells.

### CAF^R^ facilitated oxaliplatin resistance by activating the expression of the lncRNA UPK1A-AS1 in pancreatic cancer

**Fig. 3:**
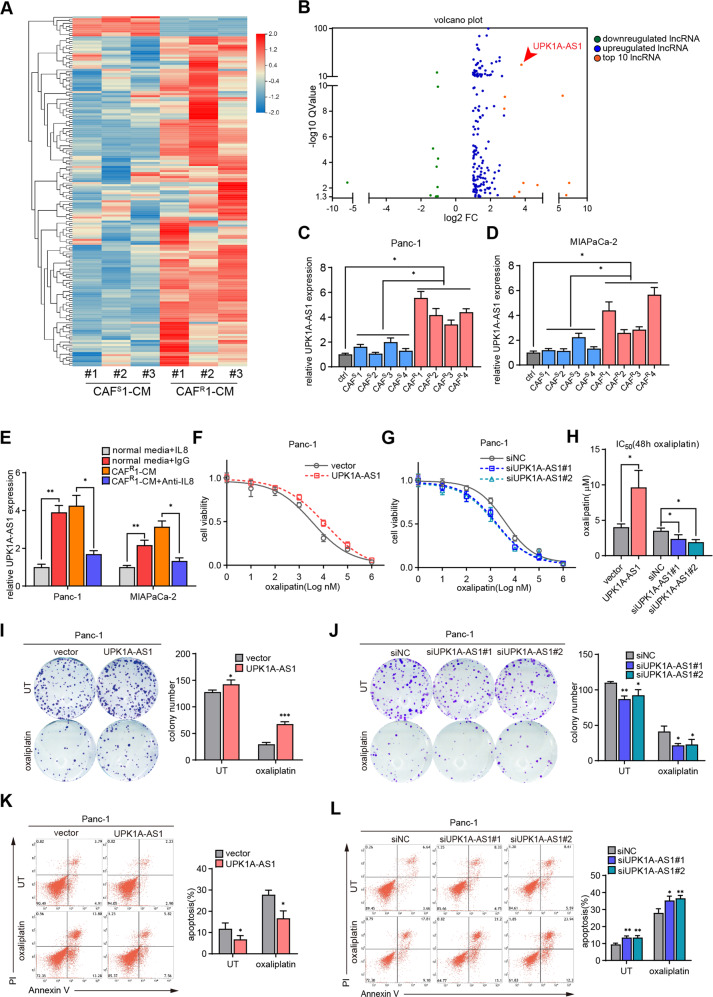
CAF^R^ facilitated oxaliplatin resistance by activating the expression of the lncRNA UPK1A-AS1 in pancreatic cancer. **A** Heatmap of the differentially expressed lncRNAs (|Log_2_^FC^ | >1, *Q* value < 0.05) in the lncRNA sequencing data of Panc-1 cells treated with CAF^S^1-CM or CAF^R^1CM. Three biological replicates of each CAF cell line were used for the RNA sequencing. **B** Volcano plot showing the differentially expressed lncRNAs between the groups. UPK1A-AS1 expression levels in Panc-1 (**C**) and MIAPaCa-2 (**D**) cells treated with CAF^S^-CM and CAF^R^-CM of four different chemosensitive and chemoresistant CAF cell lines. **E** UPK1A-AS1 expression levesl in Panc-1 and MIAPaCa-2 cells cultured under IL8 (100 ng/ml) or CAF^R^1-CM with an anti-IL8 neutralizing antibody (250 ng/ml) for 3 days. UPK1A-AS1 was overexpressed or knocked down in Panc-1 cells and oxaliplatin was given. **F–H** CCK-8 assay, (**I–J**) colony formation assay and (**K–L**) flow cytometry apoptosis analyses were performed to evaluate the chemoresistance of Panc-1 cells in each group. The results are presented as the mean ± SD of three technical replicates. *P < 0.05; **P < 0.01; ***P < 0.001.

Emerging evidence has shown that lncRNAs strongly participate in the regulation of oxaliplatin resistance [26]. To explore the mechanism underlying the regulatory role of CAFs in the chemoresistance of tumor cells, whole-transcriptome profiling (GSE183779) was performed in Panc-1 cells treated with CAF^S^1-CM and CAF^R^1-CM. Of the 173 differentially expressed lncRNAs, 163 were upregulated (Fig. 3A, B). Then, we selected the ten most significantly upregulated lncRNAs for further validation by qRT–PCR. UPK1A-AS1 was dramatically upregulated in both Panc-1 and MIAPaCa-2 cells treated with CM from 4 different CAF^R^s, compared with CAF^S^-CM (Fig. 3C, D). Moreover, similar to CAF^R^-CM, human recombinant IL8 increased the expression level of UPK1A-AS1, while CAF^R^-CM-induced activation of UPK1A-AS1 expression was attenuated by the anti-IL8 neutralizing antibodies (Figs. 3E and S4A).

To explore the influence of UPK1A-AS1 on oxaliplatin resistance in tumor cells, UPK1A-AS1 was overexpressed by transfection of a pcDNA3.1-based vector and knocked down by 2 specific siRNAs in Panc-1 and MIAPaCa-2 cells (Fig. S4B, C). An empty vector/scrambled siRNA (siNC) was used as a control. The UPK1A-AS1 induction contributed to an approximately twofold increase in the IC50 value of oxaliplatin (Figs. 3F, H, S4D, F), while the knockdown of UPK1A-AS1 significantly decreased the IC50 (Figs. 3G, H, S4E, F). Consistent with these results, the forced expression of UPK1A-AS1 promoted colony formation and reduced the suppressive effect of oxaliplatin on tumor cells (Fig. 3I and S4G). However, the UPK1A-AS1 knockdown suppressed colony formation and contributed to reduced colony survival after oxaliplatin exposure (Figs. 3J and S4H). Consistent with this result, the baseline apoptosis was reduced and oxaliplatin-induced apoptosis was abrogated by UPK1A-AS1 overexpression in tumor cells (Figs. 3K and S4I). However, the knockdown of UPK1A-AS1 enhanced baseline apoptosis and rendered the tumor cells more vulnerable to oxaliplatin-induced apoptosis (Figs. 3L and S4J).

Finally, we observed that the knockdown of UPK1A-AS1 decreased the oxaliplatin resistance of tumor cells induced by IL8 as shown by cell viability assays (Fig. S4K, L), colony formation assays (Fig. S4M, N) and Annexin-V/PI apoptosis assays (Fig. S4O, P). Based on these data, we elucidated that CAF^R^s conferred oxaliplatin resistance on tumor cells by activating UPK1A-AS1 expression in an IL8-dependent manner.

### The NF-kappa B (NF-κB) pathway was activated by IL8 from CAF^R^, and UPK1A-AS1 is a transcriptional target of p65

**Fig. 4:**
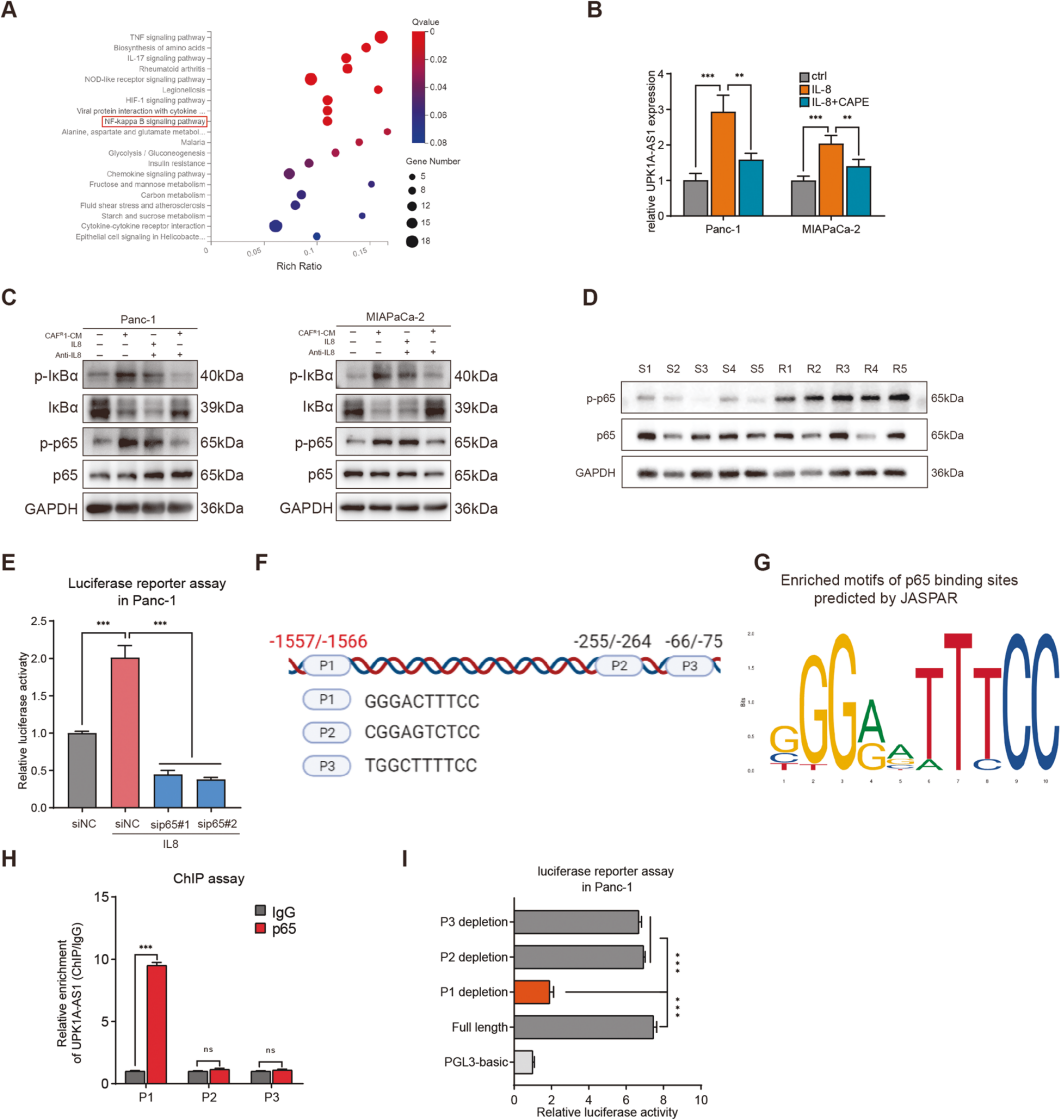
CAF^R^-derived IL8 activated the NF-κB signaling pathway to upregulate UPK1A-AS1. **A** Top 20 upregulated pathways were plotted based on the enriched gene ratio and *p* value in Panc-1 cells treated with CAF^R^1-CM compared to CAF^S^1-CM. **B** UPK1A-AS1 expression in Panc-1 and MiaPaCa-2 cells treated with IL8 (100 ng/ml) alone or IL8 and CAPE (2 μM) together for 3 days. **C** Western blot analysis of IκBα, p-IκBα, p65, and p-p65 protein expression in Panc-1 and MiaPaCa-2 cells treated with CAF^R^1-CM or IL8. A neutralizing antibody against IL8 was used to deplete IL8 in CAF^R^1-CM. **D** Western blot analysis of p65, and p-p65 protein expression in PDAC tissues from platinum-resistant patients and platinum-sensitive patients. **E** Luciferase reporter assays of Panc-1 cells transfected with a reporter plasmid containing the UPK1A-AS1 promoter, and treated with IL8 or p65 depletion. **F**, **G** A conserved p65-binding motif was predicted by JASPAR and schematic images of the potential p65 motif binding sites in the promoter of UPK1A-AS1 are shown. **H** ChIP analysis of the p65 occupancy at the promoter of UPK1A-AS1 in Panc-1 cells. **I** Luciferase reporter assays of Panc-1 cells treated with IL8 and transfected with reporter plasmids containing P1, P2 and P3 deletions in the UPK1A-AS1 promoter. The results are presented as the mean ± SD of three technical replicates. **P < 0.01; ***P < 0.001; ns no significance.

To further reveal the mechanism by which CAF^R^ activates UPK1A-AS1, the promoter regions of UPK1A-AS1 were analyzed, and the potential binding sites of several transcription factors downstream of IL8 activation, including HIF-1, p65, and STAT3 [32], in the promoter regions upstream of the transcription start site of UPK1A-AS1 were individually predicted. In addition, the KEGG pathway analysis showed enrichment of NF-κB signaling in Panc-1 cells treated with CAF^R^1-CM (Fig. 4A). Moreover, the treatment with an inhibitor of NF-κB activation (caffeic acid phenethyl ester, CAPE) significantly reversed CAF^R^-CM-induced UPK1A-AS expression (Fig. 4B). In addition, similar to the treatment with CAF^R^-CM, the treatment with IL8 successfully activated the NF-κB pathway in the tumor cells, and adding anti-IL8 antibodies attenuated the effect of CAF^R^1-CM and CAF^R^2-CM (Figs. 4C and S5A). We further confirmed the level of p-p65 in human pancreatic cancer tissues and found that the phosphorylation of p65 was upregulated in the tumor samples from the platinum-resistant patients but not in those from the platinum-sensitive patients (Fig. 4D). Luciferase reporter assays showed increased luciferase activity in the UPK1A-AS1 promoter in the IL8-treated Panc-1 cells compared with the negative control (siNC) cells, and inhibiting p65(sip65#1 and sip65#2) abrogated the above transcription-enhancing effect of IL8 (Fig. 4E). Three potential binding sites in the genomic sequences of the promoter regions of the UPK1A-AS1 locus—namely, P1, P2 and P3 (Fig. 4F, G)—were predicted with the JASPAR database. The chromatin immunoprecipitation (ChIP) assay revealed that p65-P1 directed binding to the UPKA1-AS promoter (−1557 nt/−1566 nt) in the IL8-treated Panc-1 cells (Fig. 4H). In addition, only the reporter containing the p65-P1 binding site exhibited significantly increased luciferase activity in the IL8-treated Panc-1 cells (Fig. 4I). Based on these results, we identified UPK1A-AS1 as a direct transcriptional target of p65.

### UPK1A-AS1 regulated CAF^R^-induced oxaliplatin resistance in vivo

**Fig. 5:**
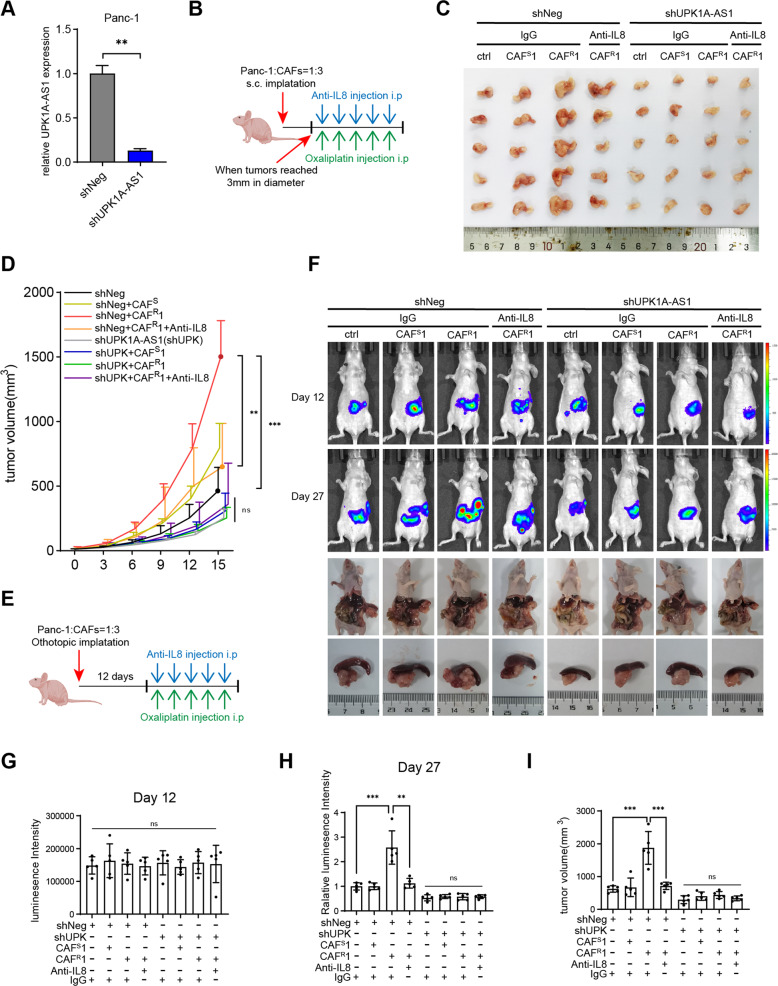
UPK1A-AS1 regulated CAFR-induced oxaliplatin resistance in vivo. **A** qRT-qPCR analysis of UPK1A-AS1 expression in Panc-1 transfected with shNeg or shUPK1A-AS1 lentivirus. **B** Once the tumors reached 3 mm in diameter, xenograft subcutaneous model receiving an anti-IL8 neutralizing antibody (20 mg/kg) and oxaliplatin (5 mg/kg) once every 3 days. **C** Representative images of xenograft subcutaneous model. **D** The changes of tumor volume were monitored and shown. **E** Xenograft orthotopic model receiving the same treatment as subcutaneous model 12 days after implantation. **F** Representative IVIS images and pancreatic tumors in orthotopic xenograft model. **G**, **H** Analysis of luminescence intensity in orthotopic xenograft model. The relative luminescence intensity = (X_day27_-X_day12_)/average(shNeg_day27_-shNeg_day12_). **I** Analysis of orthotopic tumor volume. The results are presented as the mean ± SD. *N* = 5/per group, *P < 0.05, **P < 0.01, ***P < 0.001, ****P < 0.0001. ns no significance.

We established a xenograft model of Panc-1 cells with the stable knockdown of UPK1A-AS1 (sh-UPK1A-AS1) or mock Panc-1 cells (sh-Neg) (Fig. 5A) by subcutaneous injection in nude mice. All mice were treated with oxaliplatin after the tumors reached 3 mm in diameter (Fig. 5B). Our data demonstrate that in the sh-Neg groups, the tumors in the group coinjected with CAF^R^1 grew faster than those in the control group (Panc-1 alone) or those coinjected with CAF^S^1, while the anti-IL8 neutralizing antibodies attenuated its effect. However, sh-UPK1A-AS1 markedly reduced the tumor volume and abrogated the chemoprotective effects of CAF^R^1 (Fig. 5C, D). In addition, the characteristics of animal tumor tissue were studied (Fig. S6A), less collagen deposition (Fig. S6B) and a more abundant iCAF population (Fig. S6C) were observed in the tumors coinjected with CAF^R^1. Consistently, the immunofluorescence analysis of γh2ax (Fig. S6D) and the TUNEL assay (Fig. S6E) showed that, only in the sh-Neg group, there was less DSBs and apoptosis in the tumor coinjected with CAF^R^1, which was attenuated by the anti-IL8 neutralizing antibodies. However, the chemoprotective effects of CAF^R^1 was abrogated by UPK1A-AS1 knockdown (Fig. S6D, E).

Furthermore, we analyzed the effect of UPK1A-AS1 in an orthotopic xenograft model. Luciferase-labeled Panc-1 cells were orthotopically implanted into the pancreas of nude mice, and oxaliplatin chemotherapy was administered 12 days after the pancreatic implantation (Fig. 5E). We observed that all groups initially exhibited the same level of IVIS (in vivo imaging system) signal on day 12 (Fig. 5F, G), while on day 27, in the sh-Neg groups, the mice that received the CAF^R^1 coinjection exhibited stronger fluorescence intensity in the pancreas and harbored larger tumors than the control mice or the mice received the CAF^S^1 coinjection, and this effect was attenuated by the anti-IL8 neutralizing antibodies (Fig. 5F, H, I). Conversely, sh-UPK1A-AS1 exhibited the opposite effect and abrogated the chemoprotective effects of CAF^R^1 (Fig. 5F, H, I). In addition, the characterization of the orthotopic tumor tissues of each group remained the same as that of the subcutaneous model (Fig. S6F–J). Collectively, these results support the idea that the secretion of IL8 by CAF^R^ activated the NF-κB pathway in the pancreatic cancer cells, resulting in the upregulation of UPK1-AS1 and thereby facilitating oxaliplatin resistance in pancreatic cancer.

### UPK1A-AS1 interacted with the DSB repair proteins Ku70 and Ku80

**Fig. 6:**
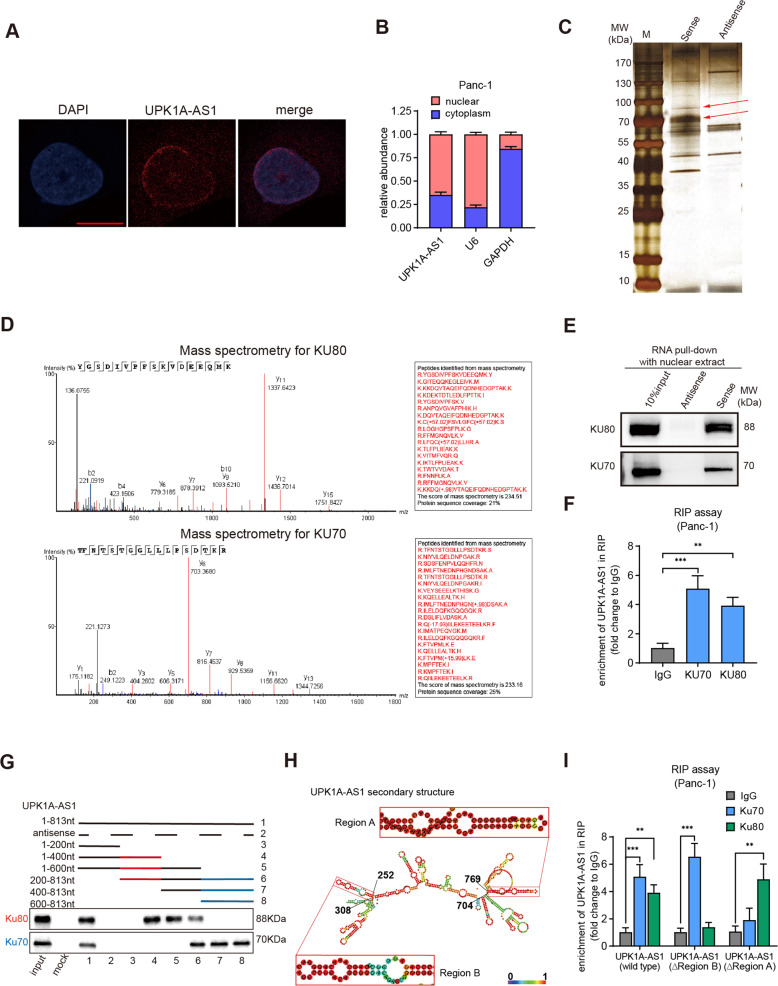
UPK1A-AS1 interacted with the DSB repair proteins Ku70 and Ku80. **A** Representative FISH images showing the cellular localization of UPK1A-AS1. The UPK1A-AS1 probe was labeled with Cy3 (red), and the nuclei were stained with DAPI (blue). Scale bar = 10 μm. **B** qRT-qPCR analysis following subcellular fractionation of UPK1AS-AS1. **C** Silver staining of UPK1A-AS1-associated nuclear proteins. Two specific bands (arrow) were excised and subjected to mass spectrometry (**D**) MS identification of UPK1A-AS1-binding proteins. **E** Western blot analysis of Ku70 and Ku80 using protein samples enriched by biotinylated UPK1A-AS1 sense and antisense RNAs. **F** Fold enrichment of UPK1A-AS1 in RNA samples precipitated with Ku70, Ku80 or IgG antibody in Panc-1 cells. **G** Western blot analysis of Ku70 and Ku80 using protein samples enriched by serial deletions of UPK1A-AS1. **H** RNAalifold predicted the secondary structure of UPK1A-AS1. The insects indicated Ku70 and Ku80 binding stem-loop structures in UPK1A-AS1. **I** Fold enrichment of UPK1A-AS1 in RNA samples precipitated with Ku70, Ku80 or IgG antibody after site-directed mutagenesis of region A and region B of UPK1A-AS1 in Panc-1 cells. The results are presented as the mean ± SD of three technical replicates. **P < 0.01; ***P < 0.001.

To further examine the subcellular localization of UPK1A-AS1, subcellular fractionation and fluorescence in situ hybridization (FISH) assays were conducted. Most UPK1A-AS1 transcripts were localized in the nuclear region (Fig. 6A, B). To explore whether UPK1A-AS1 binds nuclear protein partners involved in platinum resistance, we performed an RNA pull-down assay with nuclear extract by using a biotinylated UPK1A-AS1. The results showed that two apparent bands at ~70 kDa and 90 kDa were enriched by biotinylated UPK1A-AS1 (Fig. 6C). The apparent bands were validated as Ku70 (XRCC6) and Ku80 (XRCC5) by mass spectrometry (Fig. 6D), and further confirmed by western blotting analysis (Fig. 6E). Ku70 and Ku80 form a key element, the Ku heterodimer, to initiate rapid recognition of DSBs in the nonhomologous end joining (NHEJ) pathway [33]. The enrichment of UPK1A-AS1 by Ku70 and Ku80 was confirmed by RNA immunoprecipitation (RIP), validating the interaction between the Ku heterodimer and UPK1A-AS1 (Fig. 6F). Furthermore, the serial deletion analysis confirmed that the 600–813 nt and 200–400 nt regions of UPK1A-AS1 were indispensable for its direct interaction with Ku70 and Ku80, respectively (Fig. 6G). The sequence analysis with POSTAR2 indicated two sequence motifs and the structural preference of the binding sites for Ku70 and Ku80, which were located in the 704–769 nt (region A) and 252–308 nt (region B) regions of UPK1A-AS1 and formed a stem-loop structure (Fig. 6H). The RIP assay also showed that the enrichment of UPK1A-AS1 by Ku70 and Ku80 was attenuated after site-directed mutagenesis of regions A and B (Fig. 6I).

### The CAF^R^/IL8/UPK1A-AS1 axis regulated the efficiency of NHEJ-dependent DSB repair

**Fig. 7:**
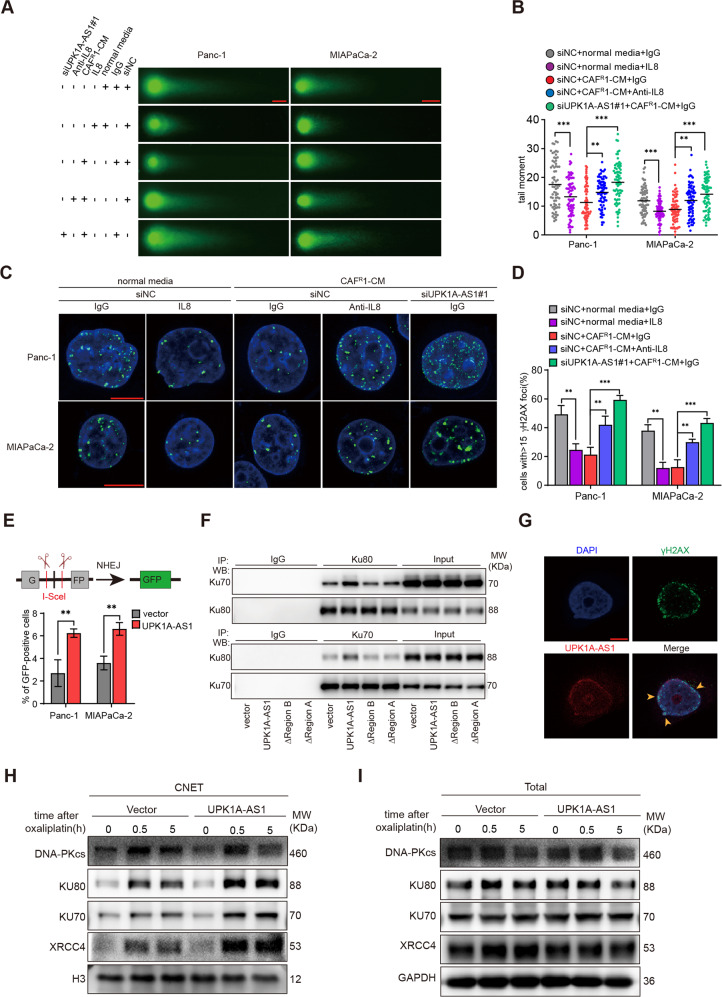
The CAF^R^/IL8/UPK1A-AS1 axis facilitated NHEJ pathway via the scaffold function of UPK1A-AS1. IL-8 (100 ng/ml), an anti-IL8 neutralizing antibody (250 ng/ml), and CAF^R^-CM were given to each group as indicated for 3 days. Panc-1 and MIAPaCa-2 cells were treated with 50 μM oxaliplatin and 30 μM oxaliplatin, respectively, in all experiments. **A** Oxaliplatin-induced DNA damage in control and UPK1A-AS1 knockdown Panc-1 and MIAPaCa-2 cells was measured by neutral comet assay. Scale bar = 10 μm. **B** Levels of oxaliplatin-induced DNA damage, quantified by the tail moment in the neutral comet assay. In total, 70 cells per group are counted. **C** Representative pictures of γH2AX-positive foci in each group. Scale bar = 10 μm. **D** Quantification of the number of γh2ax positive foci in each group. At least 40 cells per group are counted. **E** NHEJ-mediated DNA repair efficiency was measured by a pimEJ5-GFP reporter assay, in Panc-1 and MIAPaCa-2 cells transfected with control vector or UPK1A-AS1, and three technical replicates were performed. **F** Levels of Ku70 and Ku80 in the complex pulled down by Ku80 (upper panel) and Ku70 (lower panel) specific antibodies, in Panc-1 cells transfected with control vector, UPK1A-AS1 and mutated UPK1A-AS1 of region A and region B. **G** Fluorescence assessment of UPK1A-AS1 and γH2AX colocalization in Panc-1 cells. Scale bar = 5 μm. Representative images of western blotting analyses of DNA-PKcs, Ku70, Ku80 and XRCC4 in CNETs (**H**) and total proteins (**I**) of Panc-1 cells. The results are presented as the mean ± SD. **P < 0.01; ***P < 0.001.

Given that UPK1A-AS1 binds the Ku heterodimer, which is responsible for sensing DSBs in the NHEJ pathway [33], next, we examined whether DSB repair was altered by the CAF^R^/IL8/UPK1A-AS1 axis. A neutral comet assay was performed 10 h after the treatment with oxaliplatin. Shorter comet tails were observed in the human recombinant IL8- and CAF^R^-CM-treated groups, while the anti-IL8 neutralizing antibodies and UPK1A-AS1 knockdown attenuated the effect of CAF^R^1-CM and CAF^R^2-CM on reducing the lengths of the comet tails (Figs. 7A and S7A), as determined by measuring the tail moment (Figs. 7B and S7B). This finding was further confirmed by the difference in the level of γh2ax at different time points after the oxaliplatin treatment. The level of γh2ax 1 h after the oxaliplatin treatment suggested that the same amount of DSBs was generated. While the levels of γh2ax in the IL8- and CAF^R^-CM-treated groups were lower than those in the control group 10 h after the oxaliplatin induction, the γh2ax levels remained high in the anti-IL8 neutralizing antibody-treated group and UPK1A-AS1 knockdown group (Figs. S7C, D). In addition, the number of γh2ax-positive foci 10 h after the oxaliplatin induction validated this result (Figs. 7C, D and S7E, F).

To further determine whether NHEJ is the pathway affected by UPK1A-AS1, we performed pimEJ5-GFP reporter assays, in which DSBs in the reporter plasmid induced by the I-SceI endonuclease can be repaired only via NHEJ, and GFP fluorescence is observed after the repair of DSBs [34]. A significant increase in the number of GFP-positive cells was detected by flow cytometry when the tumor cells were transduced with UPK1A-AS1, indicating that NHEJ-dependent DSB repair was promoted (Fig. 7E). Taken together, these results suggest that the CAF^R^/IL8/UPK1A-AS1 axis facilitated NHEJ-dependent DSB repair.

### UPK1A-AS1 served as a molecular scaffold for Ku70 and Ku80

To further explore the mechanism by which UPK1A-AS1 regulates the NHEJ pathway, we examined the influence of UPK1A-AS1 on the interaction between Ku70 and Ku80 by co-immunoprecipitation (co-IP). In the UPK1A-AS1-overexpressing cells, there was a stronger interaction between Ku70 and Ku80 after the oxaliplatin treatment (Fig. 7F). However, site-directed mutagenesis of both regions A and B attenuated the effect of UPK1A-AS1 overexpression (Fig. 7F). Next, we examined the localization of γh2ax and UPK1A-AS1 by FISH. Surprisingly, the localization of γh2ax overlapped with that of UPK1A-AS1 (Fig. 7G). In addition, repair factors, including DNA-PKcs and XRCC4, are recruited to DSB sites following the initiation of NHEJ by the Ku heterodimer [35]. Chromatin-bound nuclear extract (CNET) was obtained after the oxaliplatin induction. Significant increases in Ku70, Ku80, DNA-PKcs, and XRCC4 binding to chromatin were observed in the UPK1A-AS1-overexpressing cells immediately and 5 h after the drug treatment (Fig. 7H), while the total levels of these factors remained stable and similar between the vector and overexpression groups (Fig. 7I). Based on these results, we elucidated that UPK1A-AS1 enhanced the interaction between Ku70 and Ku80 and was required for both effective DSB sensing and the binding of the Ku heterodimer to damaged chromatin during NHEJ-dependent DSB repair.

### Inhibition of the NHEJ pathway overcame UPK1A-AS1-induced resistance to oxaliplatin

Since UPK1A-AS1 was demonstrated to regulate oxaliplatin resistance and the NHEJ pathway, we further studied the effect of SCR7 [36], an NHEJ pathway inhibitor, on the oxaliplatin resistance conferred by UPK1A-AS1. SCR7 was used alone or in combination with oxaliplatin. In both cancer cell lines, compared to the vector-transfected cells, the UPK1A-AS1-overexpressing cells exhibited fewer γh2ax foci after the oxaliplatin exposure, as expected. The SCR7 treatment alone had almost no effect on the tumor cells. However, the SCR7 treatment successfully sensitized these resistant cells to oxaliplatin, as shown by the nonsignificant difference in the number of γh2ax foci between the vector- and UPK1A-AS1-overexpressing cells under the combination treatment with SCR and oxaliplatin (Fig. S8A–D). This result was confirmed by a western blot analysis (Fig. S8E, F). Taken together, these results again suggest the involvement of UPK1A-AS1 in DSB repair and that treatment with an NHEJ pathway inhibitor might overcome UPK1A-AS1-induced oxaliplatin resistance.

### UPK1A-AS1 overexpression was correlated with a poor prognosis in PDAC

**Fig. 8:**
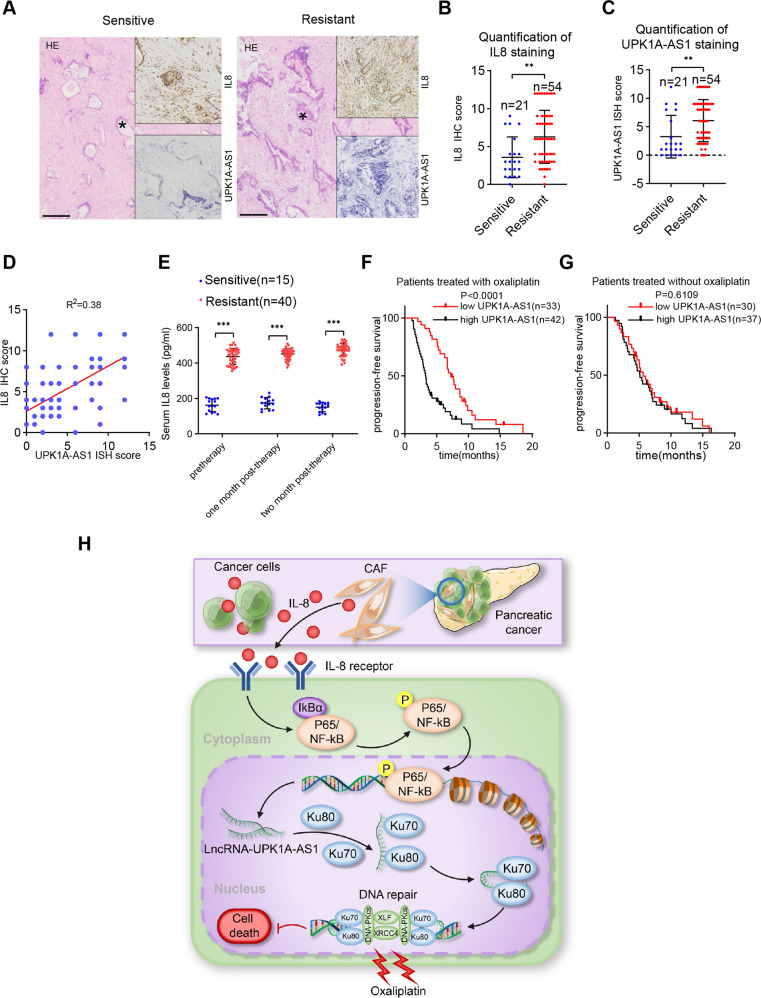
UPK1A-AS1 overexpression correlates with a poor prognosis in PDAC. **A** Representative image of ISH of UPK1A-AS1 and IHC for IL8. Scale bars = 200 μm. Quantification of IL8 (**B**) and UPK1A-AS1 (**C**) staining in tumors from platinum-sensitive and platinum-resistant patients. The results are presented as the mean ± SD of 21 and 54 biological replicates, respectively. **D** Correlation between UPK1A-AS1 expression and IL8 protein levels in PDAC tissues. **E** ELISA analysis of the serum IL-8 levels in platinum-resistant patients and platinum-sensitive patients. The results are presented as the mean ± SD. **F** Kaplan–Meier survival curves of patients who received platinum-based chemotherapy with high or low UPK1A-AS1 expression. **G** Kaplan–Meier survival curves of patients who received chemotherapy without platinum with high or low UPK1A-AS1 expression. **H** Graphical illustration of CAF^R^ derived IL8 mediating UPK1A -AS1 activation in PDAC cells to induce oxaliplatin resistance. **P < 0.01; ***P < 0.001.

To demonstrate the clinical implications of IL8 and UPK1A-AS1 in platinum-based chemotherapy, we selected a cohort of 75 patients with advanced PDAC who received platinum-based chemotherapy. Consistent with the in vitro observations, in situ hybridization (ISH) and immunohistochemical (IHC) staining showed that the platinum-resistant patients expressed higher levels of IL8 and UPK1A-AS1 than the platinum-sensitive patients (Fig. 8A–C). In addition, IL8 expression was positively correlated with UPK1A-AS1 expression (Fig. 8D). Continuous monitoring of the serum IL8 levels in the patients undergoing platinum-based chemotherapy demonstrated that the platinum-resistant patients had higher serum IL8 levels than the platinum-sensitive patients, and that the IL8 levels in the platinum-resistant patients gradually increased (Fig. 8E). Based on the dataset collected from Sun Yat-sen Memorial Hospital and Guangdong Province People’s Hospital, the Kaplan–Meier analysis indicated that the patients with higher UPK1A-AS1 expression had a shorter PFS than those with lower UPK1A-AS1 expression (Fig. 8F). Interestingly, importantly, there was no significant difference in PFS regardless of the UPK1A-AS1 level in the patients who received other chemotherapeutic regimens without platinum (Fig. 8G). Collectively, these findings indicate that CAF^R^ regulates the NHEJ pathway via IL8 paracrine-dependent activation of UPK1A-AS1 expression to induce platinum resistance in pancreatic cancer (Fig. 8H).

## Discussion

Chemotherapeutic resistance is a great challenge in the treatment of pancreatic cancer patients. Emerging evidence suggests that the TME confers innate resistance to chemotherapy [37]. As the major cell population in the tumor stroma, CAFs are largely responsible for the increased chemoresistance in PDAC. Recently, we found that CAFs promote gemcitabine resistance in pancreatic cancer via the TGF-β1/SMAD2/3 pathway and ABCC1 transactivation [38] and revealed that epigenetic modification mechanisms drive gemcitabine resistance in PDAC via LLGL1-associated phosphorylation of ER signaling pathway components [39]. However, prior to this research, knowledge regarding how lncRNAs in the TME mediate platinum resistance in PDAC was limited. Here, we identified that a higher iCAF density after platinum-based chemotherapy was associated with a worse prognosis in pancreatic cancer patients and that primary CAFs isolated from samples from chemoresistant patients conferred oxaliplatin resistance on pancreatic cancer cell lines. Furthermore, using antibody microarrays of CAFs from pancreatic cancer patients who received platinum-based chemotherapy, we demonstrated that paracrine IL8 is essential for CAF-induced oxaliplatin resistance in PDAC cells. Mechanistically, CAF-derived IL8 modulated the NF-κb/UPK1A-AS1 axis, thereby further enhancing the efficiency of NHEJ repair of DNA damage in pancreatic cancer cells to confer platinum resistance. To the best of our knowledge, this report is the first to provide insight into lncRNA-mediated epigenetic crosstalk between CAFs and pancreatic cancer cells to mediate oxaliplatin resistance, and our findings emphasize that the IL8/UPK1A-AS1 axis may constitute a novel therapeutic target for overcoming platinum resistance in pancreatic cancer patients.

Contrast studies have revealed that CAFs may play opposite tumorigenic roles in cancer development and are derived from pancreatic stellate cells, resting fibroblasts and bone marrow-derived precursors, indicating diversity and heterogeneity in CAF fate and function [40]. To identify a platinum chemotherapy-associated CAF subset in human pancreatic cancer, we compared the cytokine profiles of CAFs from tumors that were sensitive or resistant to platinum-based chemotherapy. A CAF subset with a high level of IL8 secretion that was particularly abundant in chemoresistant tumors was identified. This subset of CAFs was found to induce oxaliplatin resistance in pancreatic cancer cells by CM treatment in vitro and in a coinjection orthotopic xenograft model in vivo. Consistent with our findings, Jing et al. showed that IL8 derived from CAFs from patients with advanced gastric cancer was associated with a poor response to platinum-based neoadjuvant chemotherapy [29]. Su et al. further revealed that a new highly protumorigenic subset of CAFs promoted chemoresistance in cancer cells by upregulating the expression of IL8 and IL-6, which formed a protective layer surrounding cancer stem cells [28]. In the present study, we found that the serum IL8 level in pancreatic cancer patients with resistance to platinum-based chemotherapy was higher than that in chemosensitive patients, and that the administration of platinum-based adjuvant chemotherapy resulted in more significant differences in the IL8 level between the two groups of patients. Our data suggest that the serum level of IL8 may indicate both intrinsic and acquired chemoresistance and may be a potential marker to evaluate the response to platinum-based chemotherapy in pancreatic cancer. The association between the CAF subtypes and platinum resistance in pancreatic cancer has not been reported. We revealed for the first time that CAFs from patients with platinum-resistant pancreatic cancer are mainly characterized as iCAF subtype, suggesting that the precise targeting of iCAFs may facilitate to the improvement in platinum resistance in pancreatic cancer. Interestingly, Giulia et al. provided a novel idea that targeting iCAFs may be beneficial in the following two ways: first, depleting iCAFs could directly reduce the secretion of inflammatory factors, including IL-6and LIF factors, thereby eliminating their effect on the activation of the JAK/STAT pathway in PDAC cells, and second, it may be possible to induce a suppressive effect on PDAC by converting the iCAF subtype to the myCAF subtype [41]. Our study emphasizes that the precise targeting of iCAFs also provides an opportunity to improve platinum resistance via the inhibition of IL8-induced DSB repair in PDAC cells. Exploring and confirming whether improvement in platinum resistance in the future can be combined with the inhibition of iCAF subtype agents, including JAK inhibitors or some activators of TGF-β signaling, requires further research. Whether platinum-based regimens combined with inhibitors of iCAF subtype, such as JAK inhibitors or TGF-β signaling agonists, can improve the prognosis of pancreatic cancer patients requires further exploration. Interestingly, in our cohort of 75 PDAC patients, we found germline mutations BRCA2 or PALB2 in 4 patients (Table 1), all of whom were platinum-sensitive, which is consistent with previous finding [17, 42, 43]. It is thought-provoking that whether the targeting of iCAFs combined with platinum-based therapy and PARP inhibitors can benefit pancreatic cancer patients with germline mutations requires further exploration.

To explore the role of the lncRNAs underlying the regulatory effects of heterogeneous CAF populations on platinum-based chemoresistance in tumor cells, we identified differentially expressed lncRNAs in pancreatic cancer cells treated with CAF^S^-CM and CAF^R^-CM. We identified an IL8-induced lncRNA, UPK1A-AS1, essential for the ability of CAFs to confer oxaliplatin resistance on PDAC cells both in vitro and in vivo. Recently, UPK1A-AS1 was revealed to play an oncogenic role in hepatocellular carcinoma and protect tumor cells against cisplatin toxicity by mediating the nuclear translocation of EZH2 and competing with miR-138-5p endogenously [44]. In contrast, Yuree et al. determined that UPK1A-AS1 functions as a tumor inhibitor by enhancing the mRNA stability of UPK1A under hypoxic conditions in lung and bladder carcinoma cells [45]. An analysis of RNA-seq data in TCGA datasets revealed the tissue- or disease-specific context of UPK1A-AS1 [44], indicating that the molecular functions of UPK1A-AS1 may exert diverging effects in an organ-dependent manner. In particular, our work is the first to determine the role of UPK1A-AS1 in TME-mediated platinum resistance in PDAC. In addition to revealing the prognostic value of UPK1A-AS1 in PDAC, we discovered that UPK1A-AS1 may serve as a biomarker for the prediction of the response to platinum-based chemotherapy in PDAC patients.

Accumulating studies have substantiated that IL8 drives chemoresistance in various cancers. Recently, two preclinical studies demonstrated the mechanisms by which IL8 mediates platinum resistance by transactivating ATP-binding cassette transporters, i.e., ABCB1 in gastric cancer cells [29] and ABCB5 in tumor-initiating cells of malignant pleural mesothelioma [46]. Impressively, Wang et al. revealed a novel TME-associated mechanism by which CAF-induced IL8 mediates the phosphorylation of BRD4, leading to epigenetic remodeling and BET inhibitor resistance in colorectal cancer [47]. To the best of our knowledge, this study is the first to assess the role of lncRNAs in IL8 associated platinum resistance in PDAC.

The mechanism underlying the differential expression of UPK1A-AS1 in tumors has not been reported. In the present study, we confirmed that CAF^R^-CM is sufficient to activate NF-κB signaling via IL8, and the subsequent increase in UPK1A-AS1 expression was identified as a key driver of downstream events in NF-κB signaling. In addition, a disruption of the NF-κB pathway completely abrogated CAF^R^-CM-induced UPK1A-AS1 expression. As expected, we identified conserved κB sites of the NF-κB subunit p65 in the UPK1A-AS1 promoter, and the inhibition of endogenous p65 expression was sufficient to eliminate the promoter activity of UPK1A-AS1 in PDAC cells. In addition, the activation of UPK1A-AS1 transcription via the NF-κB signaling pathway depends on the recognition of p65 protein sites at the 1557 nt/−1566 nt promoter region. Driven mainly by inflammatory cytokines in the TME, the constitutive activation of NF-κB is widely considered a key mediator of cancer development. The key role of NF-κB signaling in the downstream regulation of lncRNAs has recently been gradually revealed. Upregulated lncRNA NKILA expression via NF-κB signaling participates in the negative feedback loop of NF-kB regulation, and thus, contributes to the metastasis and poor prognosis of breast cancer [48]. Similarly, the NF-κB signaling activation-mediated upregulation of the lncRNA LINC00665 facilitates hepatic cancer progression by mediating a positive feedback loop in the NF-κB pathway [49]. Previous studies have identified the active role of NF-κB signaling in counteracting the cytotoxic effects of platinum-based chemotherapy through the upregulation of antiapoptotic genes [50, 51], and an inhibitor of the NF-κB pathway was shown to potentiate the cytotoxicity of the chemotherapeutic agent oxaliplatin in PDAC cells [52]. In the context of the TME, our preclinical study further underlined the rationality of inhibiting NF-κB activity to reverse platinum resistance in pancreatic cancer. In addition, the findings related to the downstream regulation of UPK1A-AS1 shed light on a novel NF-κB regulatory mechanism and have clinical implications.

To gain mechanistic insight into the function of UPK1A-AS1, we studied the UPK1A-AS1-binding proteome and identified Ku70/Ku80 as protein partners involved in platinum resistance. Furthermore, we elucidated that UPK1A-AS1 is required for effective DSB sensing and for promoting the binding of the Ku heterodimer to damaged chromatin during NHEJ repair of DSBs. With the development of chemotherapeutic agents to induce DNA damage, the role of lncRNAs in DNA repair has recently been gradually revealed. DSBs represent the most significant DNA damage, and their repair involves HR and NHEJ. In eukaryotic cells, NHEJ is the preferred pathway for DSB repair due to its faster activity and versatility throughout the cell cycle [53]. In DSB repair via NHEJ, an initial synaptic complex is formed by Ku proteins after the recruitment of other NHEJ factors, including DNA-PKcs, XRCC4, XLF, APLF, and PAXX [54]. In the context of complex NHEJ, publications have noted that NHEJ factors exhibit a high binding affinity for lncRNAs, such as LINP1, SNHG12 and LRIK, and contribute to the DNA damage response [54–56]. Importantly, we provided evidence suggesting that UPK1A-AS1 serves as a molecular scaffold for Ku70 and Ku80, further functions to stabilize the binding of the Ku complex to DNA and enhances the efficiency of DSB repair. Interestingly, we confirmed the heterogeneous interactive regions between Ku70-UPK1A-AS1 and Ku80-UPK1A-AS1 complexes, both of which were indispensable for the stability of Ku70 and Ku80 and the binding of the initial synaptic complex to DNA, indicating that the versatile functions of UPK1A-AS1 may not only strengthen the Ku70/Ku80 heterodimer but also potentially enhance the crosslinking between Ku70/Ku80 heterodimers for binding DSBs during NHEJ. Collectively, these results revealed that UPK1A-AS1 serves as a scaffold for and enhancer of the NHEJ machinery and confers platinum resistance on PDAC cells, which could be explored as a targetable vulnerability.

In summary, we demonstrated a TME-mediated mechanism that enhances the efficiency of NHEJ repair of DNA damage in pancreatic cancer cells and contributes to resistance to platinum-based chemotherapy. In this context, IL8-induced UPK1A-AS1 functions as a key factor in the crosstalk between CAFs and cancer cells, supporting the urgent need for the development of rational strategies that target the IL8/UPK1A-AS1 axis to overcome platinum resistance in pancreatic cancer patients.

## Material and methods

### Patients and clinical sample

Tumor biopsy samples were collected from 75 patients with advanced PDAC who underwent first-line platinum-based chemotherapy at the Sun Yat-Sen Memorial Hospital and Guangdong Provincial People’s Hospital between 2016 and 2021. The baseline characteristics of the enrolled patients were showed in Table 1. None of the patients had received other chemotherapies, radiotherapy, immunotherapy or targeted therapy prior to the first-line platinum-based chemotherapy. Specifically, patients received platinum-based chemotherapy regimens of mFOLFIRINOX (oxaliplatin 60 mg/m^2^, irinotecan 150 mg/m^2^, and leucovorin 400 mg/m^2^ on day 1 and 46 h continuous infusion of 5-FU 2400 mg/m^2^ on days 1–2 every 2 weeks), or GemOx (gemcitabine 1000 mg/m^2^ on day 1 and day 8, and oxaliplatin 100 mg/m^2^ on day1 every 3 weeks), or GP (gemcitabine 1000 mg/m^2^ on day 1 and day 8, and cisplatin 75 mg/m^2^ on day 1 every 3 weeks). Therapeutic effects were evaluated according to the Response Evaluation Criteria in Solid Tumors [RECIST], version 1.1 [57]. Four months after the start of chemotherapy as the evaluation point, Complete Response (CR) and Partial Response (PR) were classified as chemosensitive, while Stable Disease (SD) and Progressive Disease (PD) were classified as chemoresistant. PFS was defined as the time interval from the date of chemotherapy to the date of disease progression event occurrence. All related procedures were performed with the approval of the Ethical Committee of the indicated hospitals.

### Immunofluorescence

Using paraffin-embedded samples, a PANO four-plex IHC kit (0001100020, PANOVUE, Beijing, China) was used for multiple immunofluorescence assays according to the manufacturer’s protocol. The antibodies used in this study are listed in Table S2.

Using cells, immunofluorescence was performed as previously described [58]. In brief, appropriate numbers of cells were grown on confocal dishes the day before the experiments. Panc-1 and MIAPaCa-2 cells were treated with 50 μM oxaliplatin and 30 μM oxaliplatin for 1 h and then harvested at the indicated time points. After fixation and permeabilization, the slides were incubated with antibodies against γH2AX overnight at 4 °C. Subsequently, the washed slides were incubated with Alexa Fluor 488-conjugated secondary antibodies and the nuclei were counterstained with DAPI. Finally, the immunofluorescence signals were detected by a confocal fluorescence microscope (Carl Zeiss AG, Germany). The antibodies used in this study are listed in Table S2.

### Cell lines and cell culture

The human pancreatic cancer cell lines Panc-1 and MIAPaCa-2 were purchased from ATCC (American Type Culture Collection, Rockville, MD, USA). All cells were maintained in DMEM supplemented with 10% fetal bovine serum (FBS, BI, Israel) and 1% penicillin/streptomycin and cultured at 37 °C in humidified air with 5% CO_2_.

### Primary human CAFs isolation and culture

CAFs were isolated from fresh PDAC samples by using a Human Tumor Dissociation Kit (130-095-929, Miltenyi Biotec, Germany). Briefly, the tissues were minced and digested into single-cell suspensions. After filtration with 70 mm cell strainers, the stromal fraction was collected by centrifugation at 250 *g* for 5 min and incubated with DMEM and 15% FBS. Magnetic-activated cell sorting with anti-FSP (fibroblast-specific protein) was used to purify the primary human CAFs isolated as indicated above.

### Flow cytometry

The cells were fixed and incubated with antibodies against CD31-FITC, CD45-PE/Cy7, and CD326 (EPCAM)-PE. T cells, endothelial cells and epithelial cells were used as positive controls. The antibodies used in this study are listed in Table S2.

### Conditional medium preparation

The CAFs were refreshed with DMEM and cultured for another 24 h once they reached 70% confluency. The Conditioned medium was collected, followed by centrifugation at 3000 *rpm* for 10 min. Then the conditioned medium was filtered with a 0.22 μm sterile filter and stored at −80 °C for further usage. An anti-IL8 neutralizing antibody and CAPE (S7414, Selleck, Shanghai, China) was added to each group. The antibodies used in this study are listed in Table S2.

### Cell counting kit-8 assay

Cells from different groups were seeded in 96-well plates at a density of 3000 cells per well in sextuplicate. After 24 h, the cells were incubated with a gradient concentration of oxaliplatin for 48 h. The cell viability was assessed by a Cell Counting Kit-8 assay (K1018, APExBIO, USA) according to the manufacturer’s instruction. The absorbance was measured at 450 nm with a multiwell plate reader (Spark, Tecan).

### Colony formation assay

After pretreated as indicated, ~500 tumor cells were dispersed evenly into six-well plates and allowed to attach for 24 h. Then Panc-1 and MIAPaCa-2 cells were treated with fresh complete medium containing 4 μM oxaliplatin and 2 μM oxaliplatin respectively. Twenty-four hours later, the culture medium was replaced by fresh complete medium and the cells were cultured for 2 weeks. The cells were fixed in 4% paraformaldehyde for 15 min and dyed with 0.1% crystal violet for 10 min. The colonies were counted and photographed. The experiment was repeated three times.

### Annexin V-PI apoptosis assay

Panc-1 cells were treated with 50 μM oxaliplatin (S1224, Selleck, Shanghai, China) for 48 h. MIAPaCa-2 cells were treated with 30 μM oxaliplatin for 48 h. Cell apoptosis was detected by using an Annexin V-PI staining kit (BMS500FI, Invitrogen, USA). Briefly, the cells were harvested and resuspended in Annexin V binding buffer with FITC-conjugated Annexin V and PI dye for 15 min. Then, the cells were analyzed using flow cytometer within 1 h. The experiment was repeated three times.

### RNA sequencing and data analysis

CAF^R^1 and CAF^S^1 cell lines at passage 5 were used for mRNA profiling by RNA sequencing. The total RNA was extracted, cDNA library preparation was performed and RNA sequencing was performed by Guangzhou Huayin Health Medical Group (CO.,Ltd, Guangzhou, China). GSEA_Linux_4.0.3 (https://www.gsea-msigdb.org/gsea/index.jsp) was used to complete the enrichment analysis of the gene set.

After 72 h of treatment with CAF^S^1-CM or CAF^R^1-CM in Panc-1 cells, we performed whole-genome transcriptome profiling by RNA sequencing. The total RNA was extracted, cDNA library preparation was performed and RNA sequencing was performed by BGI Technology (Co., Ltd, Shenzhen, China). The sequencing data were filtered with SOAPnuke (v1.5.2), and the clean reads were mapped to the GRCh38.p13 reference genome using HISAT2(v2.0.4). The expression level of the genes was calculated by RSEM (v1.2.12). Then differential expression analysis was performed usingDESeq2(v1.4.5) with a *Q* value ≤ 0.05. A heatmap was drawn by pheatmap (v1.0.8). A KEGG enrichment analysis of annotated differentially expressed genes was performed by Phyper (https://en.wikipedia.org/wiki/Hypergeometric_distribution) based on a hypergeometric test.

### PCR and sequencing

The DNA extraction was performed using an IPure Cell/Blood/Animal Tissue gDNA Extraction Kit (K316, IGE Biotechnology, China) according to the manufacturer’s protocol.

KRAS (codons 12, 13 and 61), TP53 (exons 3–7) and SMAD4 (codifying exons 2–11) were studied by PCR and sequencing using the primers described in Table S1. The sequencing was performed using an ABI 3730xl Genetic Analyzer (Applied Biosystems, Foster City, CA, USA) with the same primers used for the PCR, and was analyzed with sequencing analysis software v 5.3 (Applied Biosystems, Foster City, CA, USA). Regarding CDKN2A, as the most frequent mutation is homozygous deletion [59], we only performed PCR, and thus three exons, exons 1, 2 and 3, were amplified from the DNA of cell lines and a healthy control using the primers listed in Table S1.

### Cytokine antibody array

A cytokine antibody array was performed by using a Proteome Profiler Human XL Cytokine Array Kit (ARY022B, R&D Systems, USA). In brief, the CAF medium was incubated with an array membrane overnight at 4 °C, followed by incubation with detection antibody cocktails for 2 h and streptavidin-HRP for 1 h. Cytokine dots on X-ray films were scanned.

### Enzyme-linked immunosorbent assay (ELISA)

The ELISA was performed by using IL8 ELISA kits (ab48481, Abcam, USA) according to the manufacturer’s instructions. Briefly, primary CAFs were cultured in fresh serum-free medium for 24 h. Subsequently, the supernatants were collected and used for ELISA. Each experiment was repeated at least three times.

### RNA extraction, reverse transcription, qRT–PCR and subcellular fractionation

The total RNA was extracted TRIzol reagent (Invitrogen), according to the protocol and reverse transcribed by HiScript Reverse Transcriptase (R101-01, Vazyme, China). qRT–PCR was performed using a CFX96^™^ Real-Time System (Bio–Rad, USA) with a TB Green Premix Ex TaqTM kit (RR820A, Takara, Japan) and GAPDH was used as an internal control. The sequences of the primers are listed in Table S1.

Regarding the cytoplasmic and nuclear RNA fractions, cells were extracted using a PARIS Kit (AM1921, Thermo Scientific, USA) according to the protocol. Then, the cytoplasmic and nuclear ratio were measured by qRT–PCR. U6 served as the nuclear control, and GAPDH served as the cytoplasmic control. The sequences of the primers are listed in Table S1.

### Transient cell transfection and lentivirus infection

For the transient cell transfection, once the cells reached 50% confluency in six-well plates, short interfering RNA (siRNA) oligonucleotides for the target gene and the pcDNA3.1 expression vector with the target gene purchased from Gene-Pharma (Shanghai, China) were transiently transfected into Panc-1 and MiaPaCa-2 cells using Lipofectamine 3000 (L3000150, Invitrogen, USA) following the manufacturer’s instructions. The sequences of the oligonucleotides are listed in Table S1.

For the lentivirus infection, an sh-RNA plasmid was constructed and packaged into lentivirus by Gene-Pharma (Shanghai, China). Forty-eight hours after the transfection virus infection, the cells were screened and purified using puromycin (A3740, APExBIO, USA) for 2 weeks.

### Western blot analysis

The total protein was extracted from the cells and tissue samples with RIPA lysis buffer. Fractionated nuclear extract and chromatin-bound nuclear extract proteins were obtained using a Subcellular Protein Fractionation Kit for Cultured Cells (78840, Thermo Scientific, USA) according to the manufacturer’s instructions.

A western blot analysis was performed as previously described [60]. In brief, protein was electrophoretically resolved on a denaturing SDS-polyacrylamide gel and electrotransferred onto a polyvinylidene difluoride membrane. The membranes were blocked with 5% BSA for 1 h and then incubated overnight at 4 °C with specific primary antibodies, HRP-conjugated secondary antibodies were used and the bound antibodies were detected with ECL (32209, Thermo Scientific, USA). Chemi XT4 was used to visualize the expected band. The antibodies used in this study are listed in Table S2.

### Dual-luciferase reporter assays

The indicated regions of the UPK1A-AS1 promoter were directly inserted into the pGL3 luciferase reporter plasmid. A dual-luciferase reporter assay system (E1910, Promega, USA) was used to detect the luciferase activities.

### Chromatin immunoprecipitation (ChIP) assay

A ChIP analysis was performed with an EZ-Magna ChIP Chromatin Immunoprecipitation kit (17–408, Millipore, USA) according to the manufacturer’s instructions. Briefly, the cells were sheared by sonication into 100–500 bp fragments and incubated with anti-phospho-p65 overnight at 4 °C. Then the enriched DNA fragments were isolated and purified from the beads for qRT–PCR. The antibodies used in this study are listed in Table S2.

### In vivo studies

All animal studies and experimental procedures were performed under an experimental protocol approved by the South China University Animal Ethics Committee. In brief, forty 4-week-old female nude mice were randomly divided into eight groups and subcutaneously injected with 1*10^6 Panc-1 cells. CAF^s^1/CAF^R^ were combined with Panc-1 at 1:3 ratio. Once the tumors reached 3 mm in diameter, an anti-IL8 neutralizing antibody (20 mg/kg) and oxaliplatin (5 mg/kg) were given by intraperitoneal (i.p.) injection once every 3 days. After 15 days of treatment, the xenografts were harvested, fixed in 10% formalin and embedded in paraffin for subsequent analysis. The tumor volume was measured every 3 days, and calculated with the following formular: volume (mm^3^)= (width^2^ × length)/2.

Orthotopic xenograft tumor models were generated with 1*10^5 luc-Panc-1 cells in the same groups and treated as subcutaneous models. Twelve days after the implantation, treatment started and the first round of IVIS pictures was obtained. After 15 days of treatment, the second round of IVIS pictures was obtained, and the tumors were harvested. At each time point of the IVIS study, 150 mg/kg D-Luciferin, potassium Salt (40902ES01, Yeasen, China) were injected i.p., and orthotopic fluorescence images were detected using an in vivo FX PRO system (BRUKER Corporation, USA). In the in vivo study, the investigators were blind to the group allocation when assessing the outcome at each time point.

### Fluorescence in situ hybridization (FISH)

The distribution of UPK1A-AS in tumor cells was detected by FISH Kit (Gene Pharma, Guangzhou, China) according to the manufacturer’s instructions. In brief, the cells were fixed and then hybridized with Cy3-labeled UPK1A-AS1 probes overnight at 37 °C. DAPI was used to stain the nuclei. The fluorescence signals were captured by confocal microscopy (Carl Zeiss AG, Germany). The sequences of the probes are listed in Table S1.

### RNA pull-down assays

Biotinylated UPK1A-AS1 was synthesized by a Transcript Aid T7 High Yield Transcription Kit (K0441, Thermo Scientific, USA) according to the manufacturer’s instructions. Then, RNA pull-down assays were performed by a Magnetic RNA-Protein Pull-Down Kit (20164, Thermo Scientific, USA). In brief, biotinylated UPK1A-AS1 immobilized on streptavidin magnetic beads was incubated with nuclear extract overnight at 4 °C. Then, the bound captured protein was eluted from the magnetic beads and analyzed by MS or a western blot analysis. Silver staining was performed using Pierce Silver Stain Kit (24612, Thermo Scientific, USA) according to the instructions.

### RIP assays

RIP assays were performed using an EZ-Magna RIP kit (17–700, Millipore, USA) following the manufacturer′s instructions. In brief, cell lysates were incubated with target antibodies or negative control normal mouse IgG. Then the enriched RNA was isolated and purified from the beads for qRT–PCR. The antibodies used in this study are listed in Table S2.

### Neutral comet assay

Panc-1 and MIAPaCa-2 cells were treated with 50 μM oxaliplatin and 30 μM oxaliplatin for 1 h and then harvested at the indicated time points. Neutral comet assays were performed using a Comet Assay Kit (4250-050-K, Trevigen, USA) according to the manufacturer’s instructions. The samples were stained with SYBR gold (S11494, Invitrogen, USA) and observed under an Olympus FluoView 500. The quantitation of tail DNA was performed by CASP software.

### pimEJ5-GFP reporter assay

The pimEJ5-GFP reporter assay was performed as previously described [34]. In brief, tumor cells were transfected with the pimEJ5-GFP plasmid using Lipofectamine 3000. The cells were selected with puromycin to obtain stable clones. Cells that stably expressed pimEJ5-GFP were transfected with pCBASceI (I-SceI) plasmids using Lipofectamine 3000. Forty-eight to −72 h after the transfection, the GFP-positive cells were analyzed by FACS. pimEJ5-GFP was a gift from Dr. Jeremy Stark (44026, Addgene plasmid).

### Co-Immunoprecipitation (Co-IP)

Panc-1 cells transfected with control vector, UPK1A-AS1 or mutated UPK1A-AS1 in region A and region B were treated with 50 μM oxaliplatin for 1 h. CO-IP was performed using a Pierce Co-IP Kit (26149, Thermo Scientific, USA) according to the manufacturer’s instructions. In brief, the cells were harvested and lysed in IP buffer. Ten micrograms of anti-Ku70, anti-Ku80 or control IgG were added to resin and incubated with 500 μg protein mixtures overnight at 4 °C with gentle rotation. After three washes, the proteins were extracted for a western blot analysis. The antibodies used in this study are listed in Table S2.

### Immunohistochemistry

Immunohistochemistry (IHC) was performed using paraffin-embedded samples as previously described [61]. In brief, the specimens were incubated with antibodies specific for a-SMA, IL8, or Ki-67 overnight at 4 °C after deparaffinization, rehydration and heat-induced antigen retrieval. Subsequently, the specimens were washed and incubated with secondary antibodies followed by DAB developer and hematoxylin. The antibodies used in this study are listed in Table S2.

### In situ hybridization (ISH)

A double-DIG labeled UPK1A-AS1 probe (QIAGEN, Germany) was used to detect UPK1A-AS1 expression. Briefly, after deparaffinization, rehydration, and proteinase K digestion, the specimens were hybridized with a probe at 55 °C overnight. The hybridization signal was detected by an Enhanced Sensitive ISH Detection Kit (MK1032, BOSTER, China) according to the manufacturer’s instructions. The sequences of the probes are listed in Table S1.

### Quantitative analysis of IHC and ISH

The staining scores of IL8 and UPK1A-AS1 were determined based on both the staining intensity and number of positive cells. The scoring of the staining intensity was as follows: 0 (no staining), 1 (light), 2 (intermediate) and 3 (strong). The scoring of the proportion of UPK1A-AS1 positive cells was as follows: 1 (<25%), 2 (25–50%), 3 (50–75%) and 4 (75–100%). The final score was obtained by multiplying the staining intensity and proportion of positively stained cells. The expression of IL8 and UPK1A-AS1 was evaluated by the final score, with a cutoff point of <4 versus ≥4.

### Bioinformatic analysis

The secondary structure of UPK1A and the binding motifs of Ku70 and Ku80 were acquired using RNAalifold (http://rna.tbi.univie.ac.at/cgi-bin/RNAWebSuite/RNAalifold.cgi) and POSTAR2 (http://lulab.life.tsinghua.edu.cn/postar2), respectively.

### Statistical analysis

Five biological replicates were used in the animal study, and all other experiments adopted three biological replicates. The sample sizes were determined to ensure adequate power to detect a prespecified effect size based on the results of a preliminary investigation and experiment. All statistical analyses were performed using SPSS 12.0 software (IBM Corp, USA) and GraphPad Prism version 8.0 (GraphPad Software, USA). Student’s *t* test or one-way analysis of variance (ANOVA) was used for the comparisons of two or multiple groups, respectively. *R*^*2*^ was adopted to analyze the relationship between the IL8 IHC score and UPK1AS-AS1 ISH score in the PDAC specimens The survival curves were analyzed by using the Kaplan–Meier method with a log-rank test. All error bars represent the mean ± SD. *p* < 0.05 was considered statistically significant.

## Supplementary information

Supplementary Material

## Data Availability

The datasets used in the current study are available from the corresponding author upon reasonable request. The whole-genome transcriptome profile of the CAF-CM-treated Panc-1 cells and the mRNA profile of the CAF cell lines are available in the GEO database (GEO accession numbers: GSE183779, GSE192907).

## References

[1] Ho WJ, Jaffee EM, Zheng L (2020). The tumour microenvironment in pancreatic cancer - clinical challenges and opportunities. Nat Rev Clin Oncol.

[2] Ozdemir BC, Pentcheva-Hoang T, Carstens JL, Zheng X, Wu CC, Simpson TR (2014). Depletion of carcinoma-associated fibroblasts and fibrosis induces immunosuppression and accelerates pancreas cancer with reduced survival. Cancer Cell.

[3] Ohuchida K, Mizumoto K, Murakami M, Qian LW, Sato N, Nagai E (2004). Radiation to stromal fibroblasts increases invasiveness of pancreatic cancer cells through tumor-stromal interactions. Cancer Res.

[4] Hirata E, Sahai E (2017). Tumor Microenvironment and Differential Responses to Therapy. Cold Spring Harb Perspect Med.

[5] Biffi G, Tuveson DA (2021). Diversity and Biology of Cancer-Associated Fibroblasts. Physiol Rev.

[6] Moffitt RA, Marayati R, Flate EL, Volmar KE, Loeza SG, Hoadley KA (2015). Virtual microdissection identifies distinct tumor- and stroma-specific subtypes of pancreatic ductal adenocarcinoma. Nat Genet.

[7] Elyada E, Bolisetty M, Laise P, Flynn WF, Courtois ET, Burkhart RA (2019). Cross-Species Single-Cell Analysis of Pancreatic Ductal Adenocarcinoma Reveals Antigen-Presenting Cancer-Associated Fibroblasts. Cancer Disco.

[8] Hosein AN, Huang H, Wang Z, Parmar K, Du W, Huang J (2019). Cellular heterogeneity during mouse pancreatic ductal adenocarcinoma progression at single-cell resolution. JCI Insight.

[9] Öhlund D, Handly-Santana A, Biffi G, Elyada E, Almeida AS, Ponz-Sarvise M (2017). Distinct populations of inflammatory fibroblasts and myofibroblasts in pancreatic cancer. J Exp Med.

[10] Dominguez CX, Muller S, Keerthivasan S, Koeppen H, Hung J, Gierke S (2020). Single-Cell RNA Sequencing Reveals Stromal Evolution into LRRC15(+) Myofibroblasts as a Determinant of Patient Response to Cancer Immunotherapy. Cancer Disco.

[11] Biffi G, Oni TE, Spielman B, Hao Y, Elyada E, Park Y (2019). IL1-Induced JAK/STAT Signaling Is Antagonized by TGFβ to Shape CAF Heterogeneity in Pancreatic Ductal Adenocarcinoma. Cancer Disco.

[12] Park W, Chawla A, O’Reilly EM (2021). Pancreatic cancer: a review. JAMA.

[13] Grossberg AJ, Chu LC, Deig CR, Fishman EK, Hwang WL, Maitra A (2020). Multidisciplinary standards of care and recent progress in pancreatic ductal adenocarcinoma. CA Cancer J Clin.

[14] Li B, Pei G, Yao J, Ding Q, Jia P, Zhao Z (2021). Cell-type deconvolution analysis identifies cancer-associated myofibroblast component as a poor prognostic factor in multiple cancer types. Oncogene.

[15] Ham IH, Lee D, Hur H (2021). Cancer-Associated Fibroblast-Induced Resistance to Chemotherapy and Radiotherapy in Gastrointestinal Cancers. Cancers (Basel).

[16] Reiss KA, Mick R, O’Hara MH, Teitelbaum U, Karasic TB, Schneider C (2021). Phase II Study of Maintenance Rucaparib in Patients With Platinum-Sensitive Advanced Pancreatic Cancer and a Pathogenic Germline or Somatic Variant in BRCA1, BRCA2, or PALB2. J Clin Oncol.

[17] Golan T, Hammel P, Reni M, Van Cutsem E, Macarulla T, Hall MJ (2019). Maintenance Olaparib for Germline BRCA-Mutated Metastatic Pancreatic Cancer. N Engl J Med.

[18] Hato SV, Khong A, de Vries IJ, Lesterhuis WJ (2014). Molecular pathways: the immunogenic effects of platinum-based chemotherapeutics. Clin Cancer Res.

[19] Turner N, Tutt A, Ashworth A (2004). Hallmarks of ‘BRCAness’ in sporadic cancers. Nat Rev Cancer.

[20] Wattenberg MM, Asch D, Yu S, O’Dwyer PJ, Domchek SM, Nathanson KL (2020). Platinum response characteristics of patients with pancreatic ductal adenocarcinoma and a germline BRCA1, BRCA2 or PALB2 mutation. Br J Cancer.

[21] Sakai W, Swisher EM, Karlan BY, Agarwal MK, Higgins J, Friedman C (2008). Secondary mutations as a mechanism of cisplatin resistance in BRCA2-mutated cancers. Nature.

[22] Olive KP, Jacobetz MA, Davidson CJ, Gopinathan A, McIntyre D, Honess D (2009). Inhibition of Hedgehog signaling enhances delivery of chemotherapy in a mouse model of pancreatic cancer. Science.

[23] Guillen DN, Sanz-Pamplona R, Berdiel-Acer M, Cimas FJ, Garcia E, Goncalves-Ribeiro S (2019). Noncanonical TGFbeta Pathway Relieves the Blockade of IL1beta/TGFbeta-Mediated Crosstalk between Tumor and Stroma: TGFBR1 and TAK1 Inhibition in Colorectal Cancer. Clin Cancer Res.

[24] Wang W, Kryczek I, Dostal L, Lin H, Tan L, Zhao L (2016). Effector T Cells Abrogate Stroma-Mediated Chemoresistance in Ovarian. Cancer Cell.

[25] Unfried JP, Marin-Baquero M, Rivera-Calzada A, Razquin N, Martin-Cuevas EM, de Braganca S (2021). Long noncoding RNA NIHCOLE promotes ligation efficiency of DNA double-strand breaks in hepatocellular carcinoma. Cancer Res.

[26] Xie W, Chu M, Song G, Zuo Z, Han Z, Chen C, et al. Emerging roles of long noncoding RNAs in chemoresistance of pancreatic cancer. Semin Cancer Biol. 2020. 10.1016/j.semcancer.2020.11.004 33207266

[27] Yin F, Zhang Q, Dong Z, Hu J, Ma Z (2020). LncRNA HOTTIP Participates in Cisplatin Resistance of Tumor Cells by Regulating miR-137 Expression in Pancreatic Cancer. Onco Targets Ther.

[28] Su S, Chen J, Yao H, Liu J, Yu S, Lao L (2018). CD10(+)GPR77(+) Cancer-Associated Fibroblasts Promote Cancer Formation and Chemoresistance by Sustaining Cancer Stemness. Cell.

[29] Zhai J, Shen J, Xie G, Wu J, He M, Gao L (2019). Cancer-associated fibroblasts-derived IL-8 mediates resistance to cisplatin in human gastric cancer. Cancer Lett.

[30] Wilson TR, Fridlyand J, Yan Y, Penuel E, Burton L, Chan E (2012). Widespread potential for growth-factor-driven resistance to anticancer kinase inhibitors. Nature.

[31] Feng H, Liu K, Shen X, Liang J, Wang C, Qiu W (2020). Targeting tumor cell-derived CCL2 as a strategy to overcome Bevacizumab resistance in ETV5(+) colorectal cancer. Cell Death Dis.

[32] Waugh DJ, Wilson C (2008). The interleukin-8 pathway in cancer. Clin Cancer Res.

[33] Fell VL, Schild-Poulter C (2015). The Ku heterodimer: function in DNA repair and beyond. Mutat Res Rev Mutat Res.

[34] Fan L, Xu S, Zhang F, Cui X, Fazli L, Gleave M (2020). Histone demethylase JMJD1A promotes expression of DNA repair factors and radio-resistance of prostate cancer cells. Cell Death Dis.

[35] Downs JA, Jackson SP (2004). A means to a DNA end: the many roles of Ku. Nat Rev Mol Cell Biol.

[36] Srivastava M, Nambiar M, Sharma S, Karki SS, Goldsmith G, Hegde M (2012). An inhibitor of nonhomologous end-joining abrogates double-strand break repair and impedes cancer progression. Cell.

[37] Straussman R, Morikawa T, Shee K, Barzily-Rokni M, Qian ZR, Du J (2012). Tumour micro-environment elicits innate resistance to RAF inhibitors through HGF secretion. Nature.

[38] Wei L, Lin Q, Lu Y, Li G, Huang L, Fu Z (2021). Cancer-associated fibroblasts-mediated ATF4 expression promotes malignancy and gemcitabine resistance in pancreatic cancer via the TGF-beta1/SMAD2/3 pathway and ABCC1 transactivation. Cell Death Dis.

[39] Zhu YX, Li CH, Li G, Feng H, Xia T, Wong CH (2020). LLGL1 Regulates Gemcitabine Resistance by Modulating the ERK-SP1-OSMR Pathway in Pancreatic Ductal Adenocarcinoma. Cell Mol Gastroenterol Hepatol.

[40] Kobayashi H, Enomoto A, Woods SL, Burt AD, Takahashi M, Worthley DL (2019). Cancer-associated fibroblasts in gastrointestinal cancer. Nat Rev Gastroenterol Hepatol.

[41] Biffi G, Oni TE, Spielman B, Hao Y, Elyada E, Park Y (2019). IL1-Induced JAK/STAT Signaling Is Antagonized by TGFbeta to Shape CAF Heterogeneity in Pancreatic Ductal Adenocarcinoma. Cancer Disco.

[42] Waddell N, Pajic M, Patch AM, Chang DK, Kassahn KS, Bailey P (2015). Whole genomes redefine the mutational landscape of pancreatic cancer. Nature.

[43] Golan T, Kanji ZS, Epelbaum R, Devaud N, Dagan E, Holter S (2014). Overall survival and clinical characteristics of pancreatic cancer in BRCA mutation carriers. Br J Cancer.

[44] Zhang DY, Sun QC, Zou XJ, Song Y, Li WW, Guo ZQ (2020). Long noncoding RNA UPK1A-AS1 indicates poor prognosis of hepatocellular carcinoma and promotes cell proliferation through interaction with EZH2. J Exp Clin Cancer Res.

[45] Byun Y, Choi YC, Jeong Y, Yoon J, Baek K (2020). Long Noncoding RNA Expression Profiling Reveals Upregulation of Uroplakin 1A and Uroplakin 1A Antisense RNA 1 under Hypoxic Conditions in Lung Cancer Cells. Mol Cells.

[46] Milosevic V, Kopecka J, Salaroglio IC, Libener R, Napoli F, Izzo S (2020). Wnt/IL-1beta/IL-8 autocrine circuitries control chemoresistance in mesothelioma initiating cells by inducing ABCB5. Int J Cancer.

[47] Wang W, Tang YA, Xiao Q, Lee WC, Cheng B, Niu Z (2021). Stromal induction of BRD4 phosphorylation Results in Chromatin Remodeling and BET inhibitor Resistance in Colorectal Cancer. Nat Commun.

[48] Liu B, Sun L, Liu Q, Gong C, Yao Y, Lv X (2015). A cytoplasmic NF-κB interacting long noncoding RNA blocks IκB phosphorylation and suppresses breast cancer metastasis. Cancer Cell.

[49] Ding J, Zhao J, Huan L, Liu Y, Qiao Y, Wang Z, et al. Inflammation-Induced Long Intergenic Noncoding RNA (LINC00665) Increases Malignancy Through Activating the Double-Stranded RNA-Activated Protein Kinase/Nuclear Factor Kappa B Pathway in Hepatocellular Carcinoma. Hepatology. 2020;72:1666–81. 10.1002/hep.31195 32083756

[50] Wang B, Jing T, Jin W, Chen J, Wu C, Wang M (2020). KIAA1522 potentiates TNFalpha-NFkappaB signaling to antagonize platinum-based chemotherapy in lung adenocarcinoma. J Exp Clin Cancer Res.

[51] Thakur B, Ray P (2017). Cisplatin triggers cancer stem cell enrichment in platinum-resistant cells through NF-kappaB-TNFalpha-PIK3CA loop. J Exp Clin Cancer Res.

[52] Melisi D, Xia Q, Paradiso G, Ling J, Moccia T, Carbone C (2011). Modulation of pancreatic cancer chemoresistance by inhibition of TAK1. J Natl Cancer Inst.

[53] Zhao B, Rothenberg E, Ramsden DA, Lieber MR (2020). The molecular basis and disease relevance of non-homologous DNA end joining. Nat Rev Mol Cell Biol.

[54] Wang D, Zhou Z, Wu E, Ouyang C, Wei G, Wang Y (2020). LRIK interacts with the Ku70-Ku80 heterodimer enhancing the efficiency of NHEJ repair. Cell Death Differ.

[55] Thapar R, Wang JL, Hammel M, Ye R, Liang K, Sun C (2021). Mechanism of efficient double-strand break repair by a long non-coding RNA. Nucleic Acids Res.

[56] Haemmig S, Yang D, Sun X, Das D, Ghaffari S, Molinaro R (2020). Long noncoding RNA SNHG12 integrates a DNA-PK-mediated DNA damage response and vascular senescence. Sci Transl Med.

[57] Eisenhauer EA, Therasse P, Bogaerts J, Schwartz LH, Sargent D, Ford R (2009). New response evaluation criteria in solid tumours: revised RECIST guideline (version 1.1). Eur J Cancer.

[58] Causse SZ, Marcion G, Chanteloup G, Uyanik B, Boudesco C, Grigorash BB (2019). HSP110 translocates to the nucleus upon genotoxic chemotherapy and promotes DNA repair in colorectal cancer cells. Oncogene.

[59] Hustinx SR, Hruban RH, Leoni LM, Iacobuzio-Donahue C, Cameron JL, Yeo CJ (2005). Homozygous deletion of the MTAP gene in invasive adenocarcinoma of the pancreas and in periampullary cancer: a potential new target for therapy. Cancer Biol Ther.

[60] Su D, Guo X, Huang L, Ye H, Li Z, Lin L (2020). Tumor-neuroglia interaction promotes pancreatic cancer metastasis. Theranostics.

[61] Guo X, Zhou Q, Su D, Luo Y, Fu Z, Huang L (2020). Circular RNA circBFAR promotes the progression of pancreatic ductal adenocarcinoma via the miR-34b-5p/MET/Akt axis. Mol Cancer.

