# Supplementary material for: Cancer-associated fibroblast-induced lncRNA UPK1A-AS1 confers platinum resistance in pancreatic cancer via efficient double-strand break repair
Source: Oncogene. 2022 Mar 9;41(16):2372–89. doi: 10.1038/s41388-022-02253-6 (PMC9010302; doi:10.1038/s41388-022-02253-6)
Supplement: Supplementary file 1 — Supplementary Material [file 41388_2022_2253_MOESM1_ESM.pdf]

Supplementary Material

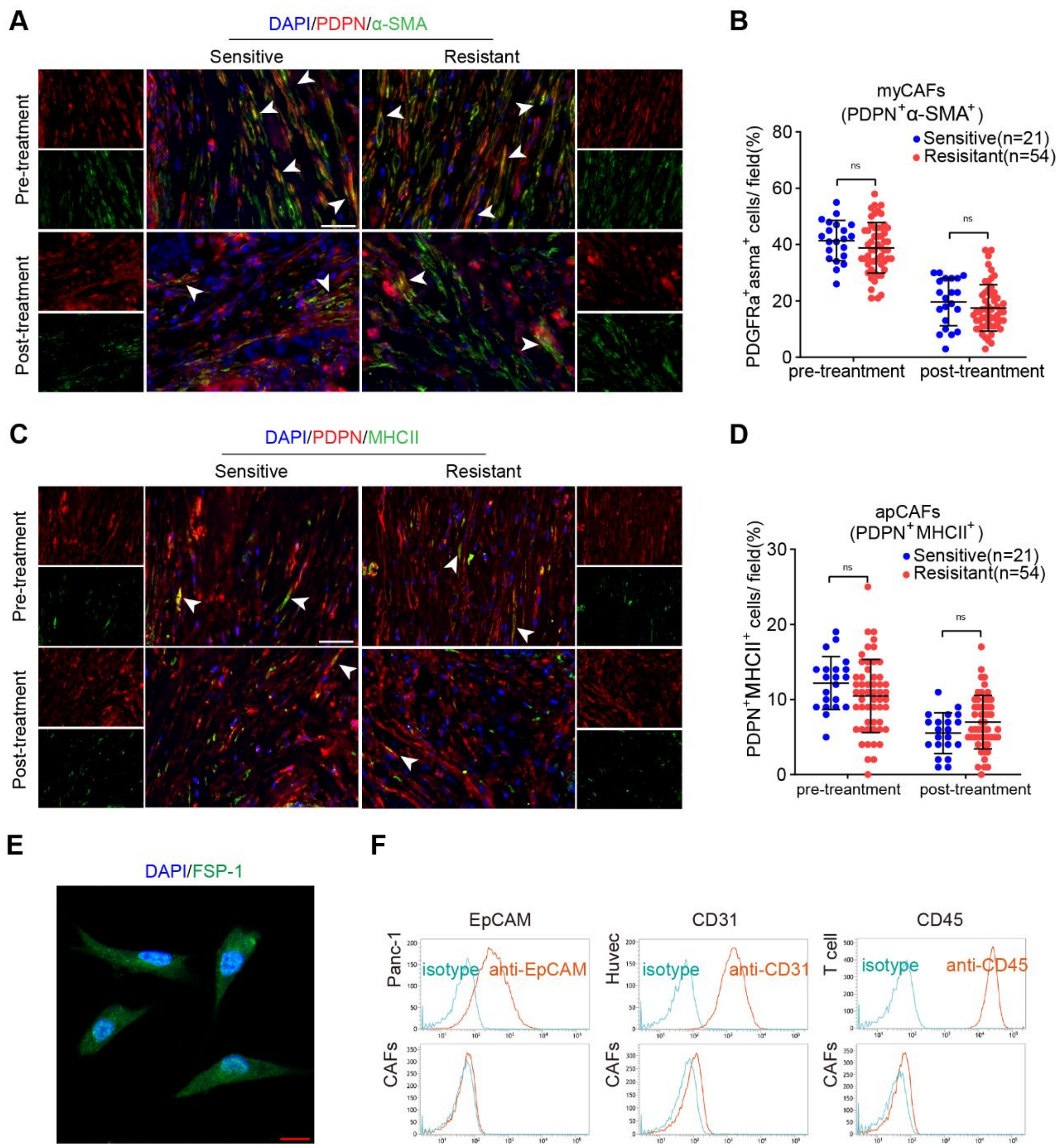

Figure S1. CAF subpopulation in tumors and identification of primary human CAFs.

(A, C) Representative immunofluorescence images of advanced pancreatic cancer biopsies before and after chemotherapy. Arrows indicate myCAFs: PDPN $^{+}$αSMA^{+} **(A)** and apCAFs: PDPN $^{+}$MHCII^{+} **(C)**, Scale bar= 50 μm. Quantification of the presence of myCAFs **(B)** and apCAFs **(D)**. **(E)** Immunofluorescence of FSP-1 in primary CAFs isolated from pancreatic cancer tissue. Scale bar=20 μm. **(F)** CAFs isolated from pancreatic cancer tissue were negative for EpCAM (epithelial marker), CD31 (endothelial marker) and CD45 (leukocyte marker), as determined by flow

cytometry. Panc-1 cells, human umbilical vein endothelial cells (HUVECs) and human T lymphocytes were used as positive controls. Images of a representative sample are shown. The results are presented as the mean ± SD. ns no significance.

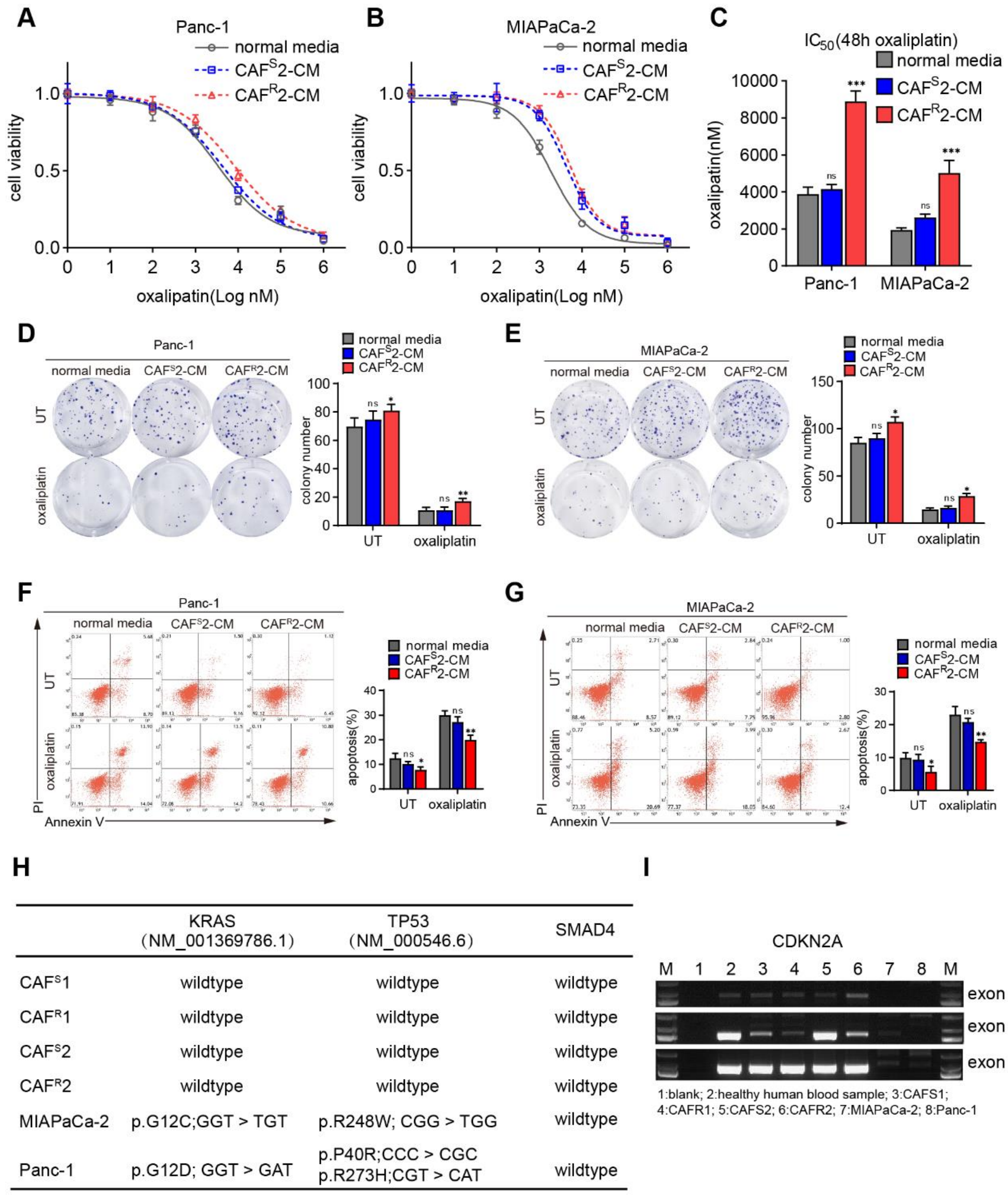

Figure S2. The in vitro function of CAF^{R}2-CM and validation of mutations in CAF cell lines.

Panc-1 and MIAPaCa-2 cells were cultured with CAF^{S}2-CM or CAF^{R}2-CM for 3 days and then subjected to the indicated experiments. **(A-C)** Cells were treated with

oxaliplatin for 48h. Cell viability was measured by CCK-8 and the IC50 value was calculated. **(D-E)** Colony formation and **(F-G)** flow cytometry apoptosis analyses were performed to evaluate the chemoresistance of pancreatic cancer cells in each group. **(H)** Mutation in CAF cell lines from Sanger sequencing data. Panc-1 and MIAPaCa-2 cells were used as positive control. **(I)** Electrophoresis results of the PCR products of exons 1, 2 and 3 of CDKN2A. Panc-1 and MIAPaCa-2 cells were used as negative controls. The results are presented as the mean ± SD of 3 technical replicates. *, P < 0.05; **, P < 0.01; ***, P < 0.001; ns no significance.

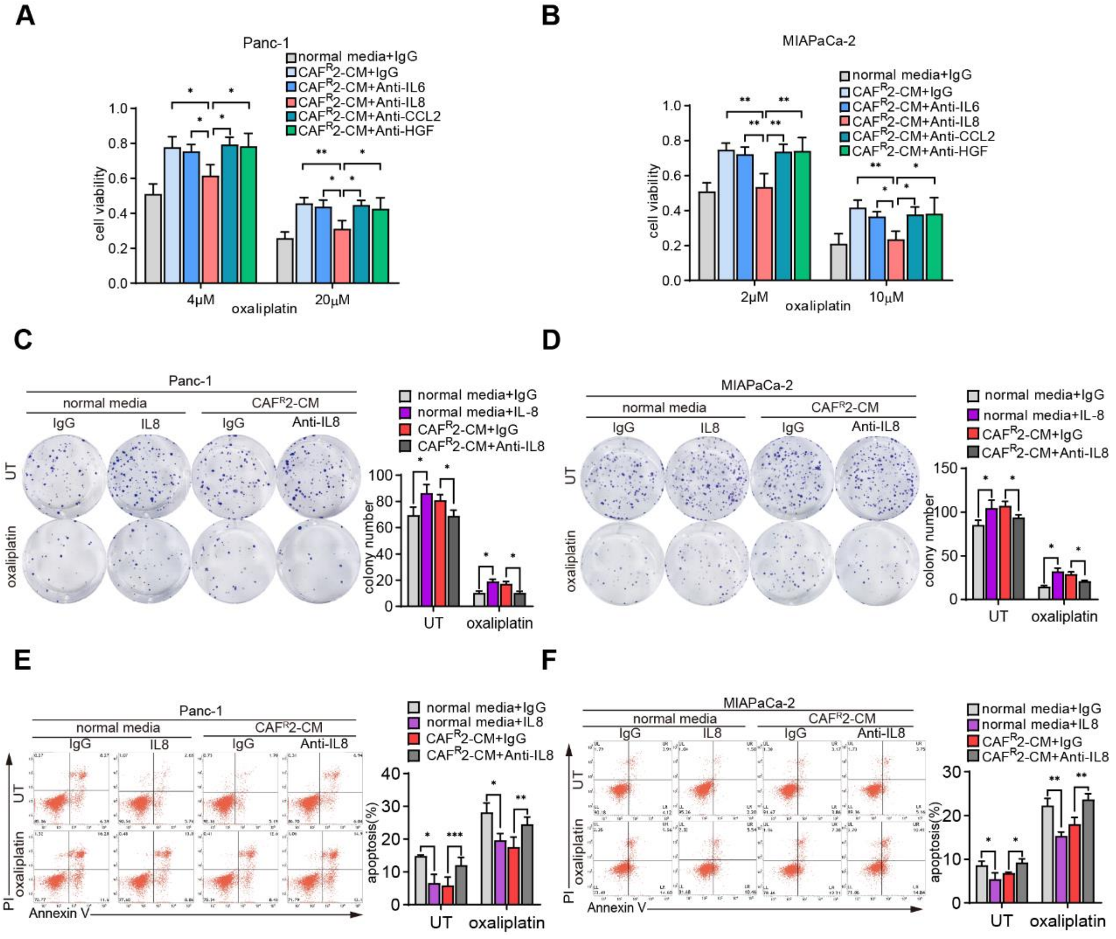

Figure S3. Paracrine IL8 was essential for CAF^R-induced oxaliplatin resistance in tumor cells.

CAF^R2-CM with different neutralizing antibodies was used to culture Panc-1 **(A)** and MIAPaCa-2 **(B)** cells for 3 days. After 48 h of oxaliplatin exposure, cell viability was measured by CCK8. Panc-1 and MIAPaCa-2 was cultured under IL8 (100 ng/ml) or

CAF^{R}2-CM with an anti-IL8 neutralizing antibody (250 ng/ml) for 3 days. Colony formation assay **(C-D)** and flow cytometry apoptosis analysis **(E-F)** were performed to evaluate the chemoresistance of pancreatic cancer cells in each group. The results are presented as the mean ± SD of 3 technical replicates. *, $P < 0.05$; **, $P < 0.01$; ***, $P < 0.001$; ns no significance.

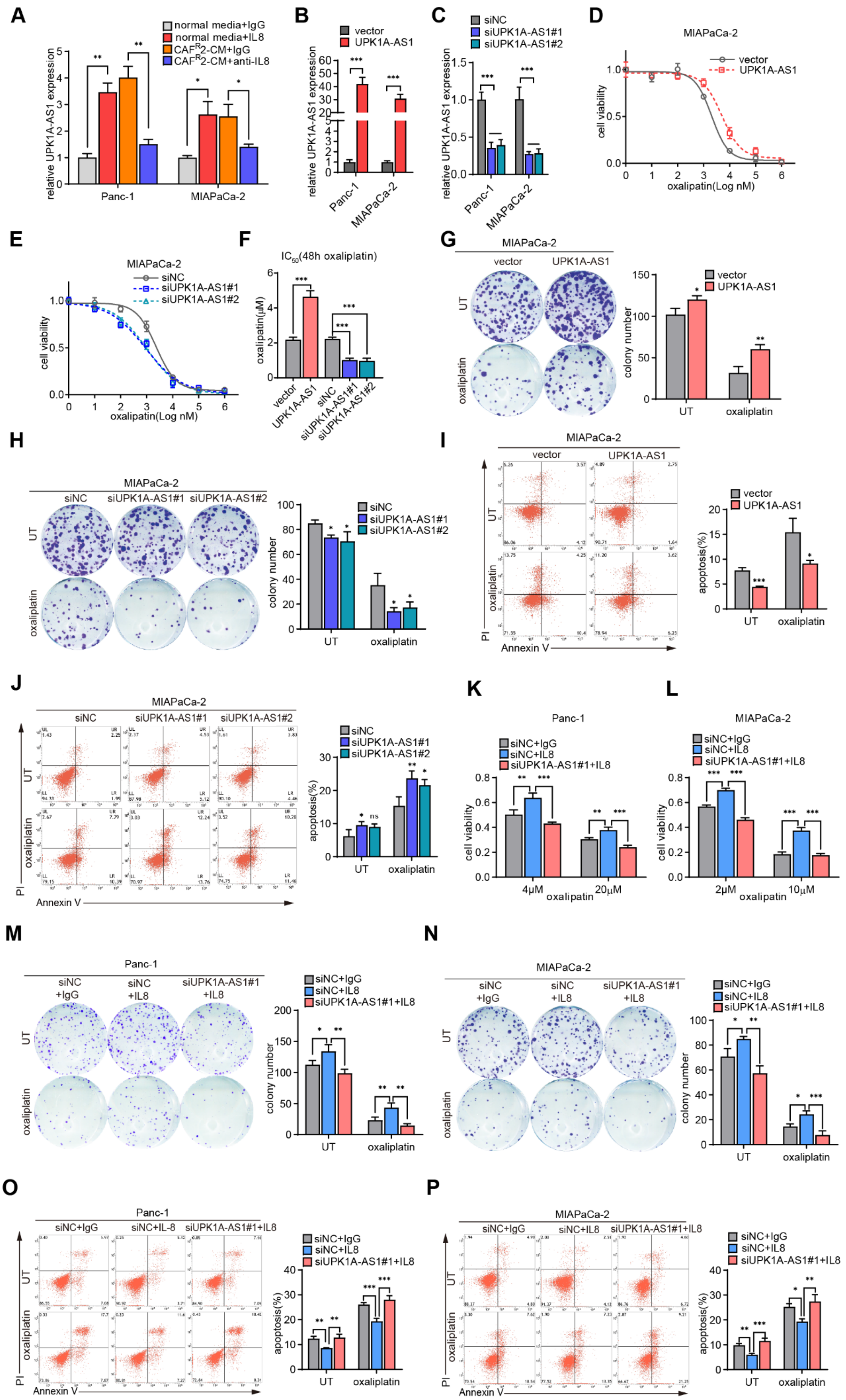
A
normal media+IgG
normal media+IL8
CAFR2-CM+IgG
CAFR2-CM+anti-IL8
relative UPK1A-AS1 expression
Panc-1
MIAPaCa-2
B
vector
UPK1A-AS1
C
siNC
siUPK1A-AS1#1
siUPK1A-AS1#2
D
MIAPaCa-2
cell viability
oxalipatin(Log nM)
E
F
IC50(48h oxaliplatin)
oxalipatin(μM)
G
UT
oxaliplatin
colony number
H
I
apoptosis(%)
PI
Annexin V
J
K
siNC+IgG
siNC+IL8
siUPK1A-AS1#1+IL8
4μM
20μM
L
2μM
10μM
M
N
O
siNC+IL-8
P

Figure S4. UPK1A-AS1 regulated oxaliplatin chemoresistance in pancreatic cancer cells.

(A) UPK1A-AS1 expression levels in Panc-1 and MIAPaCa-2 cells cultured with IL8 (100 ng/ml) or CAF^{R}2-CM with an anti-IL8 neutralizing antibody (250 ng/ml) for 3 days. **(B-C)** qRT-qPCR analysis verified the overexpression and knockdown efficiency in the Panc-1 and MIAPaCa-2. UPK1A-AS1 was overexpressed or knocked down in MIAPaCa-2 cells and oxaliplatin was given. CCK-8 assay **(D-F)**, colony formation assay **(G-H)** and flow cytometry apoptosis analyses **(I-J)** were performed to evaluate chemoresistance in each group. UPK1A-AS1 knockdown was performed in Panc-1 and MIAPaCa-2 cells. CCK-8 assay **(K-L)**, colony formation assay **(M-N)** and flow cytometry apoptosis analyses **(O-P)** revealed that the IL8 treatment (100 ng/ml for 3 days) failed to induce chemoresistance in the si-UPK1A-AS1 cells. The results are presented as the mean ± SD of 3 technical replicates. *, P < 0.05; **, P < 0.01; ***, P < 0.001; ns no significance.

A

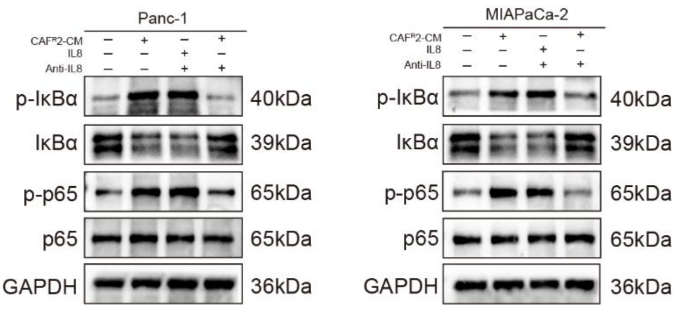

Figure S5. CAF^R-derived IL8 activated the NF-κB signaling pathway in Panc-1 and MIAPaCa-2 cells.

(A) Western blot analysis of IκBα, p-IκBα, p65, and p-p65 protein expression in Panc-1 and MiaPaCa-2 cells treated with CAF^{R}2-CM or IL8. A neutralizing antibody against IL8 was used to deplete IL8 in CAF^{R}2-CM.

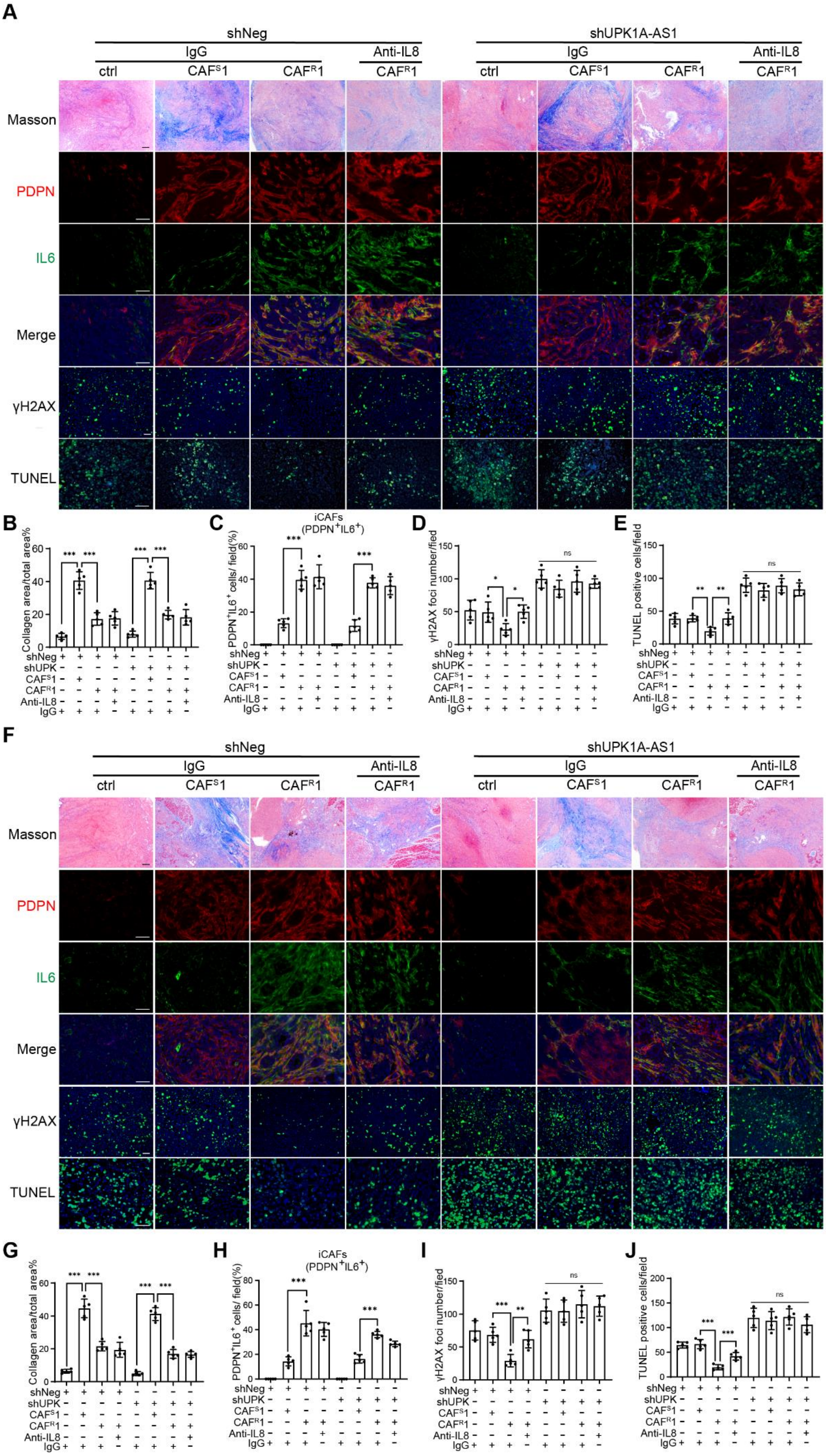
A
shNeg
shUPK1A-AS1
IgG
Anti-IL8
ctrl
CAFS1
CAFR1
Masson
PDPN
IL6
Merge
γH2AX
TUNEL
B
C
D
E
iCAFs (PDPN+IL6+)
Collagen area/total area%
PDPN+IL6+ cells/ field(%)
γH2AX foci number/fied
TUNEL positive cells/field
shNeg
shUPK
Anti-IL8
IgG
ns
F
G
H
I
J

Figure S6. Characterization of tumors tissues of animal models.

Representative images of Masson trichrome, immunofluorescence analysis of iCAFs populations and γH2AX and TUNEL assays of subcutaneous **(A)** and orthotopic model **(F)**. Black bar=200 μm, white bar=50 μm. Quantification of collage depositions **(B, G)**, the presence of iCAFs **(C, H)**, the number of γh2ax-positive foci **(D, I)** and TUNEL-positive cells **(E, J)** in each group. The results are presented as the mean ± SD. N=5/per group. *, $P < 0.05$; **, $P < 0.01$; ***, $P < 0.001$; ns no significance.

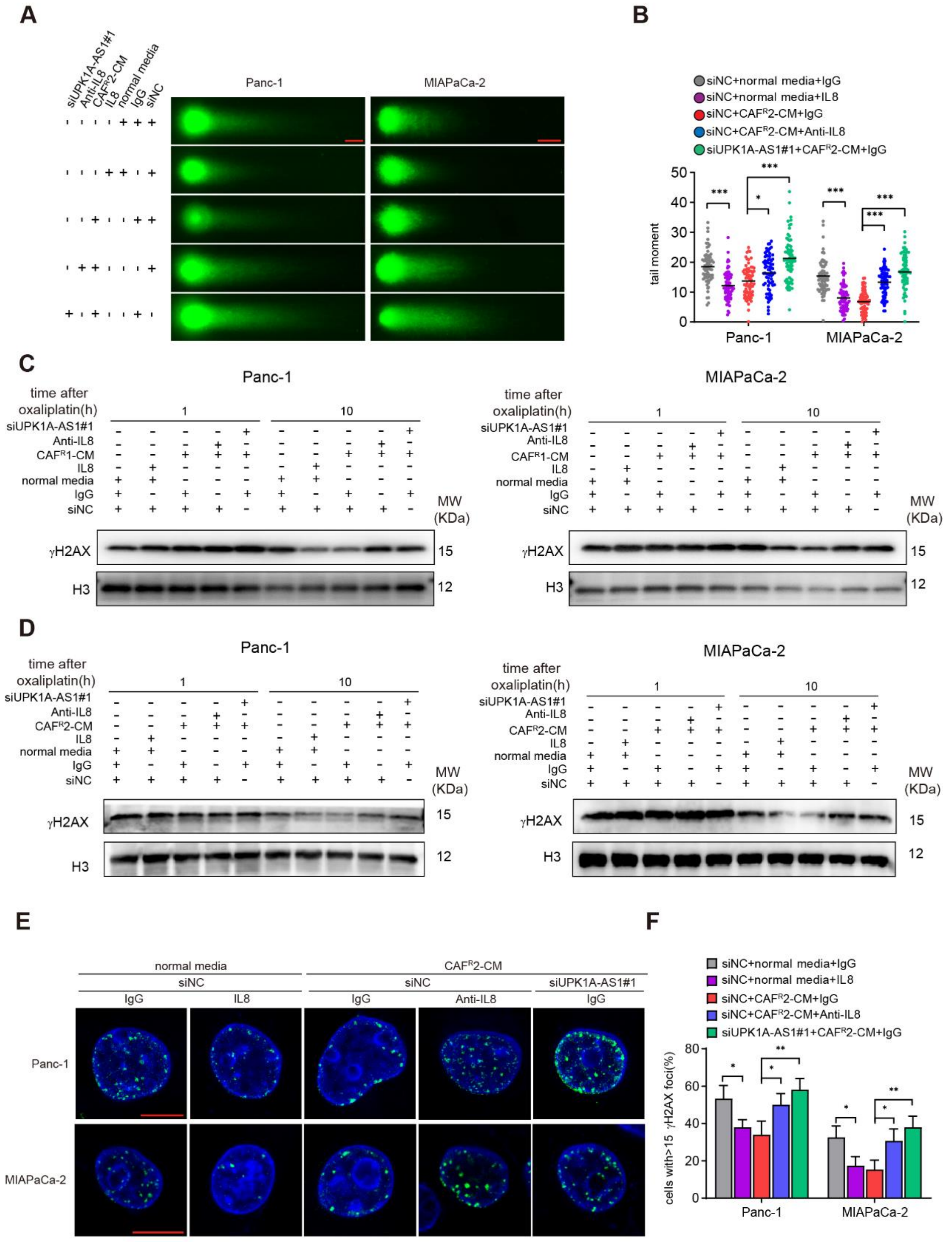

Figure S7. The CAF^{R}/IL8/UPK1A-AS1 axis attenuated the DSB induced by oxaliplatin.

IL-8 (100 ng/ml), an anti-IL8 neutralizing antibody (250 ng/ml), and CAF^{R}-CM were given to each group as indicated for 3 days. Panc-1 and MIAPaCa-2 cells were treated with 50 μM oxaliplatin and 30 μM oxaliplatin, respectively, in all experiments. **(A)**

Oxaliplatin-induced DNA damage in control and UPK1A-AS1 knockdown Panc-1 and MIAPaCa-2 cells was measured by neutral comet assay. Scale bar=10μm. **(B)** Levels of oxaliplatin-induced DNA damage, quantified by the tail moment in the neutral comet assay. In total,70 cells per group are counted. Western blot analysis of γH2AX in Panc-1 cells and MIAPaCa-2 cells treated with CAF^R1-CM **(C)** and CAF^R2-CM **(D)**. **(E)** Representative pictures of γH2AX-positive foci in each group. Scale bar=10μm. **(F)** Quantification of the number of γh2ax positive foci in each group. At least 40 cells per group are counted.

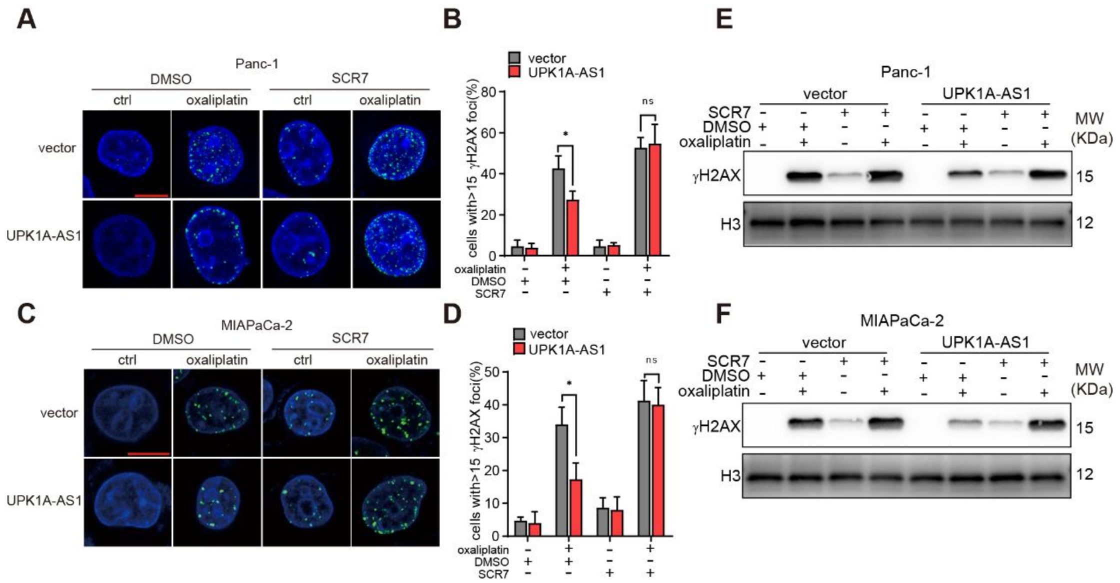

Figure S8 Inhibition of the NHEJ pathway overcame UPK1A-AS1-induced resistance to oxaliplatin

(A, C) Panc-1 and MIAPaCa-2 cells transfected with control vector or UPK1-AS1 were exposed to oxaliplatin (50 μM and 30 μM respectively) for 1 h. Then, the cells were allowed to recover for 5 h under 10 μM SCR7 or vehicle control (DMSO). Representative images of immunofluorescence of γH2AX are shown. Scale bar=10 μm. **(B, D)** Quantification of the number of γh2ax-positive foci in each group. At least 40 cells per group are counted. **(E-F)** Representative images of western blot analysis of γH2AX in each group. The results are presented as the mean ± SD. *, $P< 0.05$; ns no significance.

Table S1. Primer, probes and oligonucleotides used in the experiments.

Gene		Sequence (5'-3')	Application
UPK1A-AS1-F		AAGACCTCGCCTTCCATGAG	qRT-PCR
UPK1A-AS1-R		GCATCCCCTTTTATCCCCGA	
GAPDH-F		CCTTCCGTGTCCCCACT	qRT-PCR
GAPDH-R		GCCTGCTTCACCACCTTC	
U6-F		CTCGCTTCGGCAGCACA	qRT-PCR
U6-R		AACGCTTCACGAATTTGCGT	
IL1B-F		GGGCCTCAAGGAAAAGAATC	qRT-PCR
IL1B-R		TTCTGCTTGAGAGGTGCTGA	
IL6-F		ACTCACCTCTTCAGAACGAATTG	qRT-PCR
IL6-R		CCATCTTTGGAAGGTTCAGGTTG	
IL8-F		TAGCAAAATTGAGGCCAAGG	qRT-PCR
IL8-R		GGACTTGTGGATCCTGGCTA	
LIF-F		CCCTGGTCCCTACTCAACAA	qRT-PCR
LIF-R		CTGGACCCTGACACCCTAAA	
CSF3-F		ACGAGGGTCAGGACTGTGAC	qRT-PCR
CSF3-R		GTGACAGTGGAGGGGACACT	
COL1A1-F		GAGGGCCAAGACGAAGACATC	qRT-PCR
COL1A1-R		CAGATCACGTCATCGCACAAC	
COL1A1-F		TGGACGATCAGGCGAAACC	qRT-PCR
COL2A1-R		GCTGCGGATGCTCTCAATCT	
COL3A1-F		GCCAAATATGTGTCTGTGACTCA	qRT-PCR
COL3A1-R		GGGCGAGTAGGAGCAGTTG	
ACTG2-F		GCGTGTAGCACCTGAAGAG	qRT-PCR
ACTG2-R		GAATGGCGACGTACATGGCA	
CDH18-F		CAAAAGGGGATGGGTATGGAATC	qRT-PCR
CDH18-R		CCCGTGGTATCGTCAATGATAAA	
siUPK1A-AS1#1-sense		GCAUCUCCACGAGCAAGUUTT	siRNA
siUPK1A-AS1#1-antisense		AACUUGCUCGUGGAGAUGCTT	
siUPK1A-AS1#2-sense		GCAAAGACCUCGCCUUCCATT	siRNA
siUPK1A-AS1#2-antisense		UGGAAGGCGAGGUCUUUGCTT	
sip65#1-sense		GCACCAUCAACUAUGAUGATT	siRNA
si p65#1-antisense		UCAUCAUAGUUGAUGGUGCTT	
si p65#2-sense		GGAGUACCCUGAGGCUAUATT	siRNA
si p65#2-antisense		UAUAGCCUCAGGGUACUCCTT	
Cy3-UPK1A-AS1		CAAATGGGGAGGGAACTTGCTC	FISH
double-DIG-UPK1A-AS1		AGACCTTCCTAACCCACCGCT	ISH
KRAS	Condon 12 and 13-F	ACATGTTCTAATATAGTCAC	amplification
	Condon 12 and 13-R	CTATTGTTGGATCATATTCG	
	Condon 61-F	TTCCTACAGGAAGCAAGTAGT	amplification
	Condon 61-R	CATGGCATTAGCAAAGACTC	

TP53	Exon3-F	TGA CTG CTC TTT TCA CCC A T	amplification
	Exon3-R	AAC TCT GTC TCC TTC CTC TT	
	Exon4-F	AAC TCT GTC TCC TTC CTC TT	amplification
	Exon4-R	GCC CCA GCT GCT CAC CAT CGC TA	
	Exon5-F	TCT GAT TCC TCA CTG ATT GC	amplification
	Exon5-R	CCA GAG ACC CCA GTT GCA AA	
	Exon6-F	CCTCATCTTGGGCCTGTGTT	amplification
	Exon6-R	GCAGTAAGGAGATTCCCCGC	
	Exon7-F	CCT CTT AAC CTG TGG CTT CTC	amplification
	Exon7-R	TAA CTG CAC CCT TGG TCT CCT	
SMAD4	Exon1-F	CGTTAGCTGTTGTTTTTCACTG	amplification
	Exon1-R	ACAGTATCTGAAGAGATGGAG	
	Exon2-F	TGTATGACATGGCCAAGTTAG	amplification
	Exon2-R	CAATACTCGGTTTTAGCAGTC	
	Exon3-F	CTGAATTGAAATGGTTCATGAAC	amplification
	Exon3-R	GCCCCTAACCTCAAAATCTAC	
	Exon4-F	TTTTGCTGGTAAAGTAGTAGC	amplification
	Exon4-R	CTATGAAAGATAGTACAGTTAC	
	Exon5 and 6-F	CATCTTTATAGTTGTGCATTATC	amplification
	Exon5 and 6-R	TAATGAAACAAAATCACAGGATG	
	Exon7-F	TGAAAGTTTTAGCATTAGACAAC	amplification
	Exon7-R	TGTACTCATCTGAGAAGTGAC	
	Exon8-F	TGTTTTGGGTGCATTACATTTC	amplification
	Exon8-R	CAATTTTTTAAAGTAACTATCTGA	
	Exon9-F	TATTAAGCATGCTATACAATCTG	amplification
	Exon9-R	CTTCCACCCAGATTTCAATTC	
	Exon10-F	AGGCATTGGTTTTTAATGTATG	amplification
	Exon10-R	CTGCTCAAAGAAACTAATCAAC	
	Exon11-F	CCAAAAGTGTGCAGCTTGTTG	amplification
	Exon11-R	CAGTTTCTGTCTGCTAGGAG	
CDKN2A	Exon1-F	GCTGTTCCTGGTAGGGCCG	amplification
	Exon1-R	GCCTGGGCTAGAGACGAATTAT	
	Exon2-F	TGACACCAAACACCCCGATT	amplification
	Exon2-R	GGAAGCCTCCCCTTTTTCCG	
	Exon3-F	ATTAGACACCTGGGGCTTGTGT	amplification
	Exon3-R	ATTATTTCCCATTTGCCGCCCTG	

Table S2.Antibody used in this study.

Antibody	**Application**	**Source**
Rabbit anti-human Phospho-IκBα	WB, 1:1000	2859, CST
Rabbit anti-human IκBα	WB, 1:1000	4812, CST
Rabbit anti-human Phospho-NF-κB p65	WB, 1:1000<br>IP:1:50	3033, CST
Rabbit anti-human NF-κB p65	WB, 1:1000	8242, CST
Rabbit anti-human GAPDH	WB, 1:1000	5174, CST
Rabbit anti-human γH2AX	WB, 1:7500<br>IF, 1:250	ab81299, abcam
Rabbit anti-human Histone H3	WB, 1:2000	17168-1-AP, proteintech
Rabbit anti-human Lamin B1	WB, 1:1000	17416, CST
Mouse anti-human Ku70	WB, 1:1000<br>IP:1:100	sc-17789, santa cruz
Mouse anti-human Ku80	WB, 1:700<br>IP:1:50	sc-5280, santa cruz
Rabbit anti-human DNA PKcs	WB, 1:2000	ab32566, abcam
Rabbit anti-human XRCC4	WB, 1:1000	15817-1-AP, proteintech
IL-6 monoclonal antibody	Neutralization	554543, BD Biosciences
IL-8 monoclonal antibody	Neutralization	554726, BD Biosciences
HGF monoclonal antibody	Neutralization	AF-294-NA,R&D
CCL2 (MCP-1) monoclonal antibody	Neutralization	16-7096-81,Thermo
Rabbit anti-human IL-8	IHC/IF, 1:250	27095-1-AP, proteintech
Rabbit anti-human/mouse IL-8	IF, 1:200	A0286, ABclonal
Rabbit anti-human/mouse αSMA	IF, 1:2000	14395-1-AP, proteintech
Rabbit anti-mouse Ki67	IHC, 1:2000	ab15580, abcam
Rabbit anti-human/mouse PDGFRa	IF, 1:500	ab203491, abcam
Mouse anti-human PDPN	IF, 1:250	26981, CST
Rabbit anti-human/mouse MHCII	IF, 1:200	ab203491, abcam
Goat anti-rabbit Alexa Fluor 488	IF, 1:250	ab150077, abcam
Goat anti-mouse Alexa Fluor 555	IF, 1:250	ab150114, abcam
PE/Cy7 mouse anti-human CD45	Flow Cytometry	25-0459-42, Invitrogen
FITC mouse anti-human CD31	Flow Cytometry	555445, BD Biosciences
PE mouse anti-human CD326 (EpCAM)	Flow Cytometry	566841, BD Biosciences
